# Exploring the Altered Dynamics of Mammalian Central Carbon Metabolic Pathway in Cancer Cells: A Classical Control Theoretic Approach

**DOI:** 10.1371/journal.pone.0137728

**Published:** 2015-09-14

**Authors:** Debjyoti Paul, Abhijit Dasgupta, Rajat K. De

**Affiliations:** 1 Indian Statistical Institute, 203 B.T. Road, Kolkata 700108, West Bengal, India; 2 Machine Intelligence Unit, Indian Statistical Institute, 203 B.T. Road, Kolkata 700108, West Bengal, India; University of Nebraska Medical Center, UNITED STATES

## Abstract

**Background:**

In contrast with normal cells, most of the cancer cells depend on aerobic glycolysis for energy production in the form of adenosine triphosphate (ATP) bypassing mitochondrial oxidative phosphorylation. Moreover, compared to normal cells, cancer cells exhibit higher consumption of glucose with higher production of lactate. Again, higher rate of glycolysis provides the necessary glycolytic intermediary precursors for DNA, protein and lipid synthesis to maintain high active proliferation of the tumor cells. In this scenario, classical control theory based approach may be useful to explore the altered dynamics of the cancer cells. Since the dynamics of the cancer cells is different from that of the normal cells, understanding their dynamics may lead to development of novel therapeutic strategies.

**Method:**

We have developed a model based on the state space equations of classical control theory along with an order reduction technique to mimic the actual dynamic behavior of mammalian central carbon metabolic (CCM) pathway in normal cells. Here, we have modified Michaelis Menten kinetic equation to incorporate feedback mechanism along with perturbations and cross talks associated with a metabolic pathway. Furthermore, we have perturbed the proposed model to reduce the mitochondrial oxidative phosphorylation. Thereafter, we have connected proportional-integral (PI) controller(s) with the model for tuning it to behave like the CCM pathway of a cancer cell. This methodology allows one to track the altered dynamics mediated by different enzymes.

**Results and Discussions:**

The proposed model successfully mimics all the probable dynamics of the CCM pathway in normal cells. Moreover, experimental results demonstrate that in cancer cells, a coordination among enzymes catalyzing pentose phosphate pathway and intermediate glycolytic enzymes along with switching of pyruvate kinase (M2 isoform) plays an important role to maintain their altered dynamics.

## Introduction

Most of the cancer cells differ from normal cells with respect to their intermediary metabolism. Decades ago Otto Warburg recognized this altered metabolism in cancer cells [1, 2]. This alteration in metabolism enables the cancer cells to survive under several adverse conditions, such as hypoxia. In addition, cancer cells enable their high proliferation, progression and finally attain the stage of metastasis. Moreover, compared to the normal cells, cancer cells increase the rate of intracellular glucose import along with the fluxes through both glycolysis and pentose phosphate pathways. Again, tumor cells rely on aerobic glycolysis for energy production in the form of adenosine triphosphate (ATP) bypassing mitochondrial oxidative phosphorylation with higher production of lactate. In this scenario, uncovering the intricate altered dynamics of cancer cells may give rise to the opportunity to develop a novel diagnostic and therapeutic strategies. Thus, *in silico* metabolic pathway analysis becomes important to explore the metabolic alterations in cancer cells.

One of the most common approaches for analyzing metabolic pathways is flux balance analysis [3–5]. The methodology based on flux balance analysis can predict favourable flux distributions across the entire pathway in response to certain perturbations. It is to be mentioned here that although flux balance analysis can be used to obtain the steady state responses of the system, it is not applicable for transient responses. This is the major drawback of this method. Moreover, flux balance analysis fails to capture the key enzyme regulations and change of state (metabolites) with respect to time in response to different kinds of perturbations.

Unlike flux balance analysis, metabolic control analysis [6–9] can be used to observe both the steady-state and the transient behaviors of an individual component of a pathway. It can also be used to observe the systemic behavior of the entire network. It helps in determining the extent of control of an enzyme on both the flux of a reaction and the concentration of a metabolite with the underlying mechanisms. Through metabolic control analysis, the steps of modification to achieve some successful alterations in the reactions or metabolite production can be identified. It is helpful in the context of biotechnological (*e.g.*, large scale production of a metabolite) or clinical relevance (*e.g.*, drug therapy). In this fashion, the properties of metabolic pathways under various conditions can be well understood. However, metabolic control analysis determines only the changes of a state (metabolite) with respect to certain parameters. Moreover, it does not provide any supervisory controller to manipulate concentrations of enzymes/metabolites to attain certain objectives/needs of cells.

Thus, we have used principle of the classical control theory to explore the altered dynamics of mammalian central carbon metabolic (CCM) pathway in cancer cells. It is capable of dealing with the behavior of nonlinear dynamic systems. Metabolic pathways can be considered as a process based on classical control theory because of its nonlinearity in nature. An earlier investigation [10] shows how metabolic pathway analysis can be reformulated in the classical control theoretic framework. In this context, the authors have used some feedback linearization techniques based on classical control theory [11]. They have studied different steady states and robustness of regulated glycolysis and glycogenolysis. However, nonlinear dynamics of CCM pathway both in the normal cells and the cancer cells are still to be explored from the classical control theoretic point of view. Thus, an approach with nonlinear modeling of metabolic pathway is necessary to analyze the effects of various parameters for such a pathway. Here we have modeled metabolic pathways using the nonlinear state space modeling. Thus, the present work deals with development of a new computational methodology, based on classical control theory, for analyzing control mechanisms of metabolic pathways, both in normal and cancer cells.

In our approach, the reactions are modeled by standard Michealis Menten kinetics [12] with some modifications to incorporate feedback [13] mechanisms along with perturbations and cross talks employed in most of the reactions. We have considered various factors in the proposed model. They are the sources of different disturbances associated with each metabolite and enzyme concentration. The crosstalk between various interlinked pathways, in terms of enzyme stimulation, has a contributing factor in the responses of a whole metabolic network. Since, the disturbances/noises/perturbations are not predefined (unpredictable) in the case of a biological system, we have considered the perturbations as random varying signals. We have considered only those reactions which have control points. The control points indicate the reactions employed in regulatory activities. Here, we have developed a model mimicing the actual dynamic behavior of mammalian CCM pathway in normal cells [12].

Since cancer cells rely on aerobic glycolysis for energy (ATP) production, we have perturbed the enzymatic activities of both pyruvate dehydrogenase and pyruvate carboxylase by setting values of their initial concentrations to nearly zeros. As a result, the mitochondrial oxidative phosphorylation reduces. Furthermore, we have connected proportional-integral (PI) controller(s) with the model. We have discussed about the controllers in the following section. Here, we have tuned the PI controller(s) in such a way to produce sufficient amount of energy in the form ATP along with nicotinamide adenine dinucleotide phosphate (reduced form) (NADPH), D-ribose-5P and phosphoenolpyruvate (PEP), which help in forming cell building materials (*e.g.*, nucleotide). Thus, we have developed a model which behaves like the CCM pathway in a mammalian cancer cell [14, 15].

Simulation results demonstrate that for cancer cells, there is a coordination among enzymes catalyzing pentose phosphate pathway and intermediate glycolytic enzymes. In addition, the switching of pyruvate kinase (M2 isoform) between its two oligomeric forms, *i.e.*, active tetramer and almost inactive dimer, plays an important role for cancer cells to survive under adverse conditions, such as hypoxia. The results of the proposed model have been validated using some previous experimental results. Unlike our proposed model, previous modeling techniques based on flux balance analysis, metabolic control analysis and classical control theory have failed to capture this kind of altered dynamics in cancer cells.

## Preliminaries

Here, we are going to discuss some basic concepts on the dynamics of biochemical pathways and classical controllers.

### Dynamics of biochemical pathways

Here, we describe, in brief, dynamics of biochemical pathways in general, metabolic pathways in particular. In most of the situations, the models of dynamic systems under consideration are complex. There are several higher order and complicated mathematical formulations to represent these models. Yet they are unsuitable for modeling large scale systems, like metabolic pathways, as they are time consuming and need devices with high computational power, storage and accuracy. Instead, we can consider simplified models that are capable of capturing the main properties of the actual dynamic systems under study. Thus model reduction may be adopted for handling this kind of problems. The order of a system is simply the number of state variables necessary to describe the dynamics of the system. For analysis of the control action in metabolic pathways, we are mainly interested in those reactions which have some control mechanisms embedded in them, the reactants and/or products through which the pathway cross talks with others. If the associated parameters are altered, it is likely to affect the entire pathways. Here we are more concerned about the reactions employed in feedback inhibition of enzymes, and perturbations that can influence the dynamics of the pathways. We do not analyze all the reactions that are present in the pathways because the dynamics of a pathway is guided by some key reactions along with substrates, products, hormones and corresponding catalyzing enzymes that are employed in regulatory mechanisms.

Michaelis Menten kinetics expresses the relationship between substrates and enzymatic interactions of a metabolic pathway at a particular instant. Moreover, the equation based on Michaelis Menten kinetics describes the rate of enzymatic reactions. It relates an initial reaction rate *V* to [*X*], the concentration of a substrate *X*. The equation is given by

$$V=\frac{V_{max}\cdot \left[X\right]}{K_{m}+\left[X\right]}=\frac{K\cdot [E]\cdot \left[X\right]}{K_{m}+\left[X\right]}.$$ (1)

Here, *V*_max_ (= *K* ⋅ [*E*]) represents the maximum rate achieved by the reaction at maximum (saturating) substrate concentrations. [*E*] is the total enzyme concentration, *i.e.*, the sum of the free and substrate-bound enzyme concentrations. Michaelis constant *K*_*m*_ is the substrate concentration at which the reaction rate is half of *V*_max_. A small value of *K*_*m*_ indicates high affinity depicting that the rate of the reaction will approach more quickly to *V*_*max*_, at saturating substrate concentrations [12]. The value of *K*_*m*_ depends on concentrations of both the enzyme *E* and the substrate *X*, as well as conditions such as temperature and pH. The term *K* is the turnover number/reaction rate constant which represents maximum number of substrate molecules converted to product molecules per enzyme molecule per second. The values of the kinetic constants *K*_*m*_ and *K* used in the proposed model vary with respect to enzymes and physiological conditions. The values of *K*_*m*_ and *K* lie between 10^−7^ M and 10^−1^ M, and 1 *s* ^−1^ and 10^5^ *s* ^−1^ respectively, for the majority of enzymes [16, 17]. The values of *K*, and initial concentrations of major metabolites for CCM pathway in human erythrocytes with appropriate scaling [17] can be found in Tables 1 and 2.

**Table 1:** Reaction rate constants of major enzymes for the CCM pathway in human erythrocytes [17].

Enzyme	Value of *K*
Hexokinase	9.96 × 10^1^ *h* ^−1^
Glucose 6-phosphate isomerase	9.56 × 10^3^ *h* ^−1^
Phosphofructokinase1 (PFK1)	5.81 × 10^5^ *h* ^−1^
Aldolase	1.03 × 10^6^ *h* ^−1^
Triosephosphate isomerase	7.30 × 10^4^ *h* ^−1^
Glyceraldehyde phosphate isomerase	9.06 × 10^3^ *h* ^−1^
Phosphoglycerate kinase	6.68 × 10^3^ *h* ^−1^
Diphosphoglycerate mutase	3.44 × 10^3^ *h* ^−1^
Diphosphoglycerate phosphatase	2.6 × 10^0^ *h* ^−1^
Phosphoglycerate mutase	3.90 × 10^5^ *h* ^−1^
Enolase	7.95 × 10^4^ *h* ^−1^
Pyruvate kinase	2.69 × 10^2^ *h* ^−1^
Lactate dehydrogenase	1.02 × 10^1^ *h* ^−1^
Adenylate kinase	7.50 × 10^3^ *h* ^−1^
ATPase	3.56 × 10^−1^ *h* ^−1^
Glutathione oxidation	3.00 × 10^−2^ *h* ^−1^
Glutathione reductase	7.53 × 10^3^ *h* ^−1^
Glucose 6-phosphate dehydrogenase	3.88 × 10^3^ *h* ^−1^
6-phosphogluconate dehydrogenase	2.86 × 10^3^ *h* ^−1^
Ribose phosphate epimerase	3.77 × 10^4^ *h* ^−1^
Ribose phosphate isomerase	2.51 × 10^3^ *h* ^−1^
Phosphorybosylpyrophosphate synthetase	8.71 × 10^4^ *h* ^−1^
Transketolase1	1.68 × 10^0^ *h* ^−1^
Transaldolase	1.2 × 10^1^ *h* ^−1^
Transketolase2	6.01 × 10^1^ *h* ^−1^

**Table 2:** Initial concentrations of major metabolites for CCM pathway in human erythrocytes [17].

Metabolite	Value
*α*-D glucose 6P	0.0385 mM
*β*-D fructose 6P	0.0157 mM
*β*-D fructose 1, 6*P*_2_	0.007 mM
D-Glyceraldehyde 3P	0.0057 mM
Glycerone P	0.14 mM
Glycerate 1, 3*P*_2_	0.0005 mM
Glycerate 3P	0.0685 mM
Glycerate 2P	0.01 mM
Phosphoenolpyruvate (PEP)	0.017 mM
6-phosphogluconate	0.0049 mM
Ribulose 5-phosphate	0.016 mM
Xylulose 5-phosphate	0.016 mM
D-ribose-5P	0.018 mM
Sedoheptulose 7-phosphate	0.0199 mM
Erythrose 4-phosphate	0.0076 mM
nicotinamide adenine dinucleotide phosphate (NADP)	0.0014 mM
ATP	1.83 mM
adenosine monophosphate (AMP)	0.037 mM

Nonlinear complex systems, especially multivariate systems (*e.g.*, metabolic pathways), can be handled using state space model in a more convenient way. For a nonlinear system, the state space model is represented by

$$\dot{x}=f\left(x,u\right)$$ (2)

and

y=h(x,u), (3)

where *f* and *h* are nonlinear functions of state **x** and control input **u**. Here $\dot{x}$ represents time (*t*) derivative of **x** and **y** represents output of the system.

Let us consider a metabolic pathway comprising *m* metabolites and *n* reactions. The metabolic pathway can be represented by [3]

$$\frac{dx}{dt}=N\cdot v$$ (4)

Eq (4) represents a set of nonlinear differential equations that form the kinetic model for a (metabolic) network of coupled chemical reactions and transport processes, where **N** is the stoichiometric matrix of order *m* × *n* [18, 19]. The flux vector **v**(**x**, **z**) ∈ ℝ^*n*^ (ℝ being the set of real numbers) is a function of both metabolite concentration vector **x** ∈ ℝ^*m*^ and reaction parameters **z** ∈ ℝ^*n*^. The term **z** signifies the relevant kinetic parameters such as enzyme concentrations and catalytic efficiencies.

### Classical controllers

We have already mentioned that cancer cells alter the normal dynamics of the CCM pathway. Consequently, we have put our proposed model into such a situation that it mimics exactly the abnormal behaviour of the CCM pathway in cancer cells. In this scenario, we have used PI controllers. Here, we have introduced the concepts of controllers before going to further details of our methodology.

**Fig 1:**
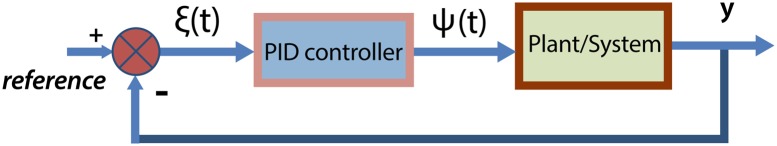
Block diagram of closed loop control system. It illustrates the role of PID controller to drive a certain output in accordance with corresponding reference input.

Proportional-Integral-Derivative (PID) controller is a standard controller in control system. It consists of three components, *i.e.*, P (proportional), I (integral) and D (derivative). The proportional component generates a signal proportional to the error signal, while the integral component creates a signal proportional to the area under the error curve. Besides, derivative component is responsible for an output control signal proportional to the rate of change of the error signal. The error signal is the deviation of the actual output generated by a plant/system from the desired output/reference input. Fig 1 illustrates how a PID controller works in a closed loop control system. The term *ξ*(*t*) represents the error signal in time domain *t*, *i.e.*, the difference between the reference input signal and the actual output *y*. *ξ*(*t*) acts as the input to the PID controller. Here, the PID controller computes both the derivative and the integral of *ξ*(*t*). The output *ψ*(*t*) (control signal) of a PID controller is governed by the following time-domain (*t*) equation,

$$\psi \left(t\right)=K_{p}\cdot \xi \left(t\right)+K_{int}\cdot \int \xi \left(t\right)\cdot dt+K_{d}\cdot \frac{d\xi (t)}{dt}$$ (5)

Here *K*_*p*_, *K*_*int*_ and *K*_*d*_ are the proportional, integral and derivative gains respectively. The control signal *ψ*(*t*) is applied to the plant to generate updated output. This process continues till the error signal *ξ*(*t*) will become very close to zero.

The derivative component of a PID controller only predicts the future errors based on linear extrapolation. In other words, it is used to predict the error curve by considering the rates of changes in various factors under consideration. Subsequently, PID controller performs well without considering the derivative control mode (*K*_*d*_ = 0). Here, it can be termed as PI controller. Similarly, P controller considers *K*_*int*_ = *K*_*d*_ = 0, while PD controller considers *K*_*int*_ = 0 only.

The majority of closed loop control systems in industry have been modeled using only the proportional and integral control modes. Proportional mode helps a closed loop control system in getting an immediate response to an error. Besides, the integral mode eliminates the long term error. Hence, derivative mode is not needed. In this scenario, existing literature does not provide any clue of such an instance where the control/regulatory mechanism occurs due to the rate of change in a metabolite rather it depends on the amount of the metabolite accumulated in a cell. Thus, it is enough to use a PI controller to model altered characteristics of CCM pathway in cancer cells.

## Method

In our model, each pathway is characterized by the following four parameters (pathway variables). We have used the same notations in the entire manuscript to denote the pathway variables.

1. I (⊂ ℝ): Set of concentrations of input metabolites, hormones and perturbations, such that $e_{i}^{I}\in I,\;\forall_{i}$, influence a metabolic pathway. Moreover, I_*s*_ ⊂ I and I_*d*_ ⊂ I such that $I_{s}\cap I_{d}=⌀$ and I_*s*_ ∪ I_*d*_ = I. Here I_*s*_ and I_*d*_ are respectively the sets of concentrations of essential metabolites/hormones uptake and undesired metabolites creating disturbances/perturbations for the metabolic pathway.
2. D (⊂ ℝ): Set of desired/reference concentrations/fluxes, such that $e_{i}^{D}\in I,\;\forall_{i}$, represent desired concentrations of products (metabolites) and fluxes of reactions as per need of the cell.
3. O (⊂ ℝ): Set of actual/resultant concentration/flux outputs, such that $e_{i}^{O}\in I,\;\forall_{i}$, represent actual/resultant concentrations of products (metabolites) and fluxes of reactions.
4. E (⊂ ℝ): Set of concentrations of enzymes, such that $e_{i}^{E}\in I,\;\forall_{i}$, represent concentrations of different enzymes catalyzing the reactions of the metabolic pathway.

According to our model, a PI controller applied to a metabolic pathway tries to minimize the error function *ϑ*_*i*_(*t*), $\forall \;e_{i}^{D}$ and $\forall \;e_{i}^{O}$ in the time domain *t*, varying 𝓔 and 𝓘. The term *ϑ*_*i*_(*t*), ∀_*i*_ is defined as

$$\vartheta_{i}\left(t\right)=|e_{i}^{D}-e_{i}^{O}|\;,\forall_{i}.$$ (6)

Here we have set the reference inputs (concentrations/fluxes) for certain metabolites/reactions in [0, 1] assuming them as needs of cancer cells. Each of them is considered as $e_{i}^{D}$. Each of actual outputs (concentrations/fluxes) for these metabolites/reactions generated by the model is represented by $e_{i}^{O}$.

**Fig 2:**
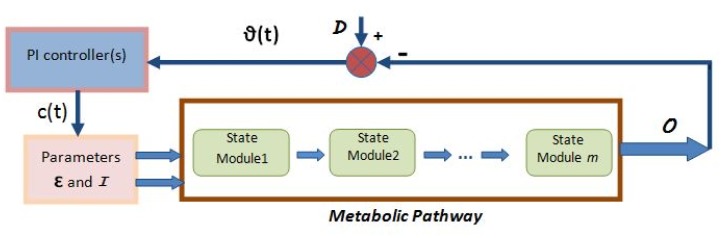
Block diagram illustrating the entire methodology. Here, metabolic pathway module (collection of the state modules) represents the normal model for CCM pathway. PI controller module drives the normal model to behave like the altered CCM pathway in cancer cells.

We have modeled each of the CCM pathway reactions in an individual module (Fig 2). Each of these modules represents a state (metabolite) variable feeding resultant state to the next module. They are connected sequentially to simulate the actual dynamics of the CCM pathway in normal cells. Thereafter, perturbing the normal model along with tuning of PI controller(s), we have tried to meet certain needs (*e.g.*, energy demand) of cancer cells. In this process, the error input *ϑ*(*t*) is applied to the PI controller(s). Besides, the outputs of the PI controller(s) affect concentrations of different input metabolites $e_{i}^{I}$ and enzymes $e_{i}^{E}$, which are responsible to meet the desired demands of the mutated cells. For example, if the mutated cells need a large amount of pyruvate concentration, the cells may try to enhance the enzymatic activities of phosphofructokinase1 (PFK1) and pyruvate kinase. Moreover, in this approach, it needs the coordination among different PI controllers based on the relative importance of the different requirements of the mutated cells.

Let us assume that the output *c*(*t*) in the time-domain *t* of a PI controller is driven by the error function *ϑ*(*t*). Thus, *c*(*t*) is governed by the following equation.

$$c\left(t\right)=K_{p}\cdot \vartheta \left(t\right)+K_{int}\cdot \int \vartheta \left(t\right)\cdot dt$$ (7)

Here *K*_*p*_ and *K*_*int*_ are the proportional and integral gains respectively. Eq (7) is just another form of Eq (5) considering *K*_*d*_ = 0. The term *c*(*t*) is responsible to alter appropriate parameters ($e_{i}^{I}$ or $e_{i}^{E}$) for the purpose of generating energy and cell building materials in cancer cells.

Thus, the proposed methodology has two major parts. One describes techniques to develop the actual dynamics of the CCM pathway in normal cells. The other deals with mimicing altered dynamics of the CCM pathway in cancer cells.

### Model for the CCM pathway in normal cells

Some basic steps are followed to develop the model for CCM pathways in normal cells. They are discussed here with appropriate examples.

#### Order reduction

**Fig 3:**
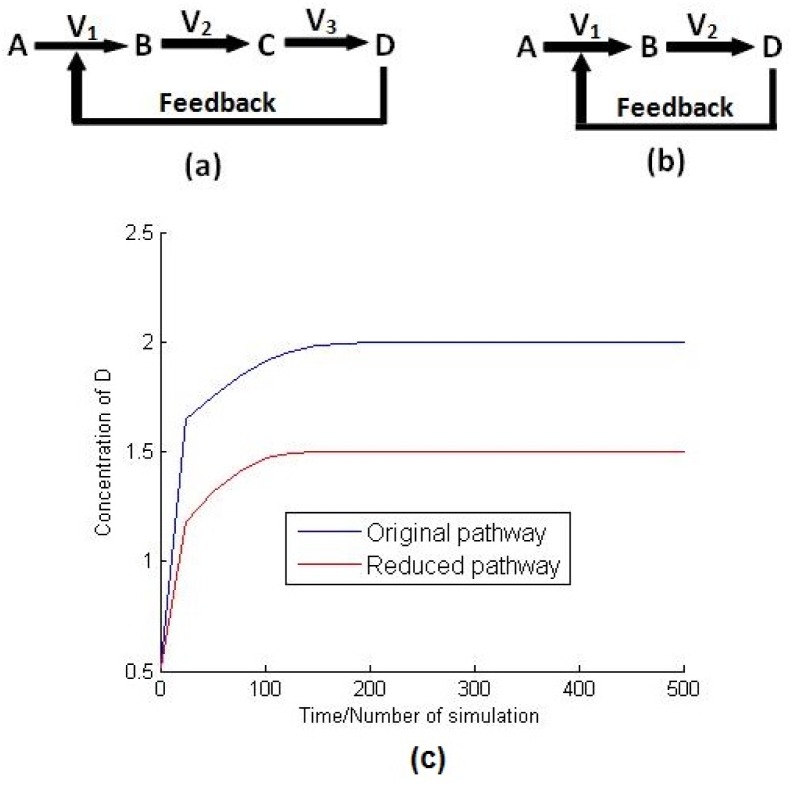
Order reduction. It represents (a) the original pathway, (b) the reduced pathway and (c) the variation of concentration of D with respect to time *t* for both of the original and reduced pathways.

Let us consider that a hypothetical pathway consists of four metabolites *A*, *B*, *C* and *D* involved in three reactions with fluxes *V*_1_, *V*_2_ and *V*_3_ as given in Fig 3(a). The enzyme catalyzing the reaction *A* → *B* is inhibited by the accumulated metabolite D. It can be assumed that metabolite D is formed directly from B in a single reaction, if C, being produced by *B* → *C*, is fully consumed by *C* → *D* (Fig 3(b)). This reduction is quite valid since there is no control mechanism employed in the reaction *B* → *C*, and C does not cross talk with other pathways. This assumption has been verified, in Fig 3(c), by simulating these two pathways, *i.e*, original pathway and reduced one using COPASI software [20]. The details of the simulation and the instances of different parameters for these two pathway models can be found in S1 Table. Although there is some amount of changes in the steady state values, Fig 3(c) depicts similar behavior of the original and the reduced pathways.

It is true that this kind of reduction introduces some amount of differences in the values of metabolite concentrations/reaction fluxes at steady state but the pattern of responses remains unaltered. However, the main motivation behind the use of such reduction technique is to reduce computational complexity. Moreover, the existing database (KEGG) includes several small steps for a reaction. For example, in the case of the reaction pyruvate → acetyl-CoA, there are three intermediate steps catalyzed by the same enzyme pyruvate dehydrogenase. They can easily be treated as a single reaction without affecting the nature of dynamic responses of the system (CCM pathway in normal cells) except introducing some amount of differences in the values of metabolite concentrations/reaction fluxes at steady state. Since, our main objective is to explore the relative effect of different key metabolites, enzymes and perturbations in a metabolic network, these differences may be neglected. They do not affect the pattern of the dynamic responses of the metabolic pathway. Besides, the system remains stable despite this reduction.

**Fig 4:**
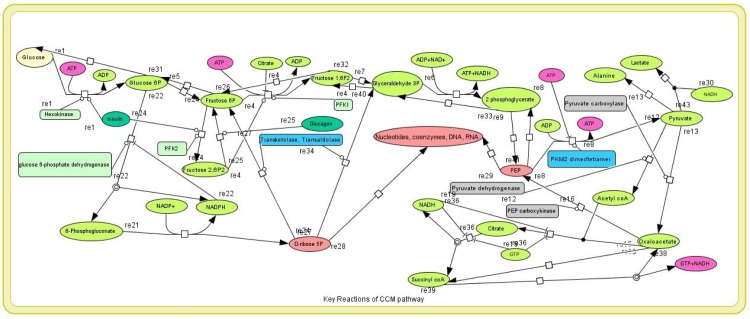
CCM pathway. It shows some key reactions of reduced CCM pathway.

We have applied the order reduction technique to reduce the order of CCM pathway (Fig 4). In this fashion, we have reduced the original CCM pathway (47 reactions in KEGG database as depicted in S2 Table) of human erythrocytes to the one with 28 reactions (S3 Table) involved in key regulatory activities. Although our proposed methodology can directly be applied to all the 47 reactions, it may not be necessary to consider all of them. The model with 28 reactions is capable enough to capture the original pattern of dynamic responses of CCM pathway. S1 Fig compares two models of the pathways consisting of 47 reactions and 28 reactions to show similar pattern of dynamic responses with variation of glucose and pyruvate kinase for both the cases. Here, we have considered all the reactions as irreversible. In other words, we have treated reversible reactions as combination of two irreversible reactions.

#### Stoichiometric matrix

**Fig 5:**
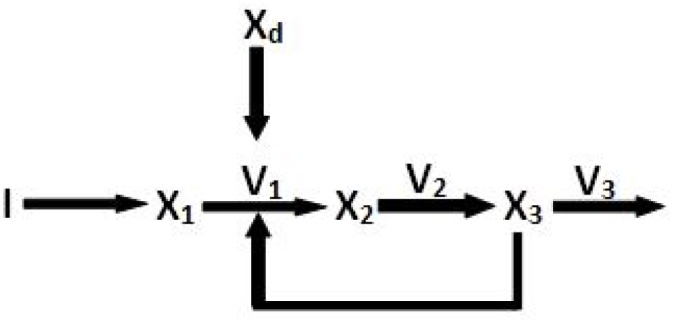
Example. Here, it is a hypothetical biochemical pathway.

Let us consider a simple hypothetical metabolic pathway with feedback inhibition as depicted in Fig 5. This pathway has three metabolites, *i.e.*, *X*_1_, *X*_2_ and *X*_3_, along with 3 reactions (*X*_1_ → *X*_2_, *X*_2_ → *X*_3_ and *X*_3_ consumption reaction) catalyzed by the enzymes *E*_1_, *E*_2_ and *E*_3_ respectively. The initial fluxes of the three reactions are *V*_1_, *V*_2_ and *V*_3_ respectively. Besides, the metabolite *X*_1_ is supplied by a separate reaction *I* → *X*_1_ catalyzed by an enzyme *E*_0_. The reaction flux *V*_1_ is inhibited by the accumulation of *X*_3_. Subsequently, there is a perturbation *X*_*d*_ which activates/inhibits the flux *V*_1_. The stoichiometric matrix of the hypothetical pathway is given by

$$N_{hypothetical}=(\begin{array}{ccc} -1 & 0 & 0\\ 1 & -1 & 0\\ 0 & 1 & -1 \end{array}).$$ (8)

Similarly, we have defined the stoichiometric matrix **N**_*CCM*_ for CCM pathway.

#### Modifying Michaelis Menten kinetic equation

As defined in Eq (1), the rate of the first reaction (*X*_1_ → *X*_2_ in Fig 5), being inhibited, is modified to incorporate feedback inhibition [13] and other different disturbances/perturbations. If *X*_1_ is consumed in a reaction with turnover number/rate constant *K*_1_ and Michaelis constant *K*_*m*_1__, which is inhibited by a substrate *X*_3_, then initial reaction rate is modified as

$$V_{1}=\frac{K_{1}\cdot \left[E_{1}\right]\cdot \left[X_{1}\right]}{\left(K_{m_{1}}+\left[X_{1}\right]\right)\left(1+F\cdot \left[X_{3}\right]\right)}$$ (9)

Suppose if *X*_3_ accelerates the reaction that consumes *X*_1_. Then the modified Michaelis-Menten kinetics becomes

$$V_{1}=\frac{\left(K_{1}\cdot \left[E_{1}\right]\cdot \left[X_{1}\right]\right)\left(1+F\cdot \left[X_{3}\right]\right)}{K_{m_{1}}+\left[X_{1}\right]}.$$ (10)

Here we have ignored the perturbation *X*_*d*_ which has been considered in Eq (13). The term F (feedback constant) determines the relative inhibition (Eq (9)) or activation strength (Eq (10)) of a metabolite/hormone. Higher the value of F, stronger is the inhibition or activation effect. We have initialized F values for different reactions with random numbers in [0, 1]. We have illustrated the significance of the feedback constant F with an example of the hypothetical metabolic pathway (Fig 5) in the following section.

#### State vector and output vector

Eq (4) can directly be mapped to the nonlinear state space Eqs (2) and (3). The resultant nonlinear state space representation of a metabolic pathway can be represented by

$$\dot{x}=N\cdot v$$ (11)

and

y=v (12)

where $\dot{x}$ represents rates of changes of the concentrations of metabolites with respect to time, and **y** represents resultant flux vector of the reactions participating in the metabolic pathway.

#### Simulink model

As depicted in Fig 5, let the flux vector be **v** = [*v*_1_ *v*_2_ *v*_3_]^*T*^ and metabolite concentration vector be **x** = [*x*_1_ *x*_2_ *x*_3_]^*T*^, while *I* is the input substrate. The term *x*_*d*_ is a perturbation which either inhibits the reaction/enzyme or activates it. Each reaction flux of *v*_1_, *v*_2_ and *v*_3_ is modeled using modified Michaelis-Menten kinetic equation (Eqs (9) and (10)).

$$v_{1}=\frac{K_{1}\cdot \left[E_{1}\right]\cdot \left[x_{1}\right]}{\left(K_{m_{1}}+\left[x_{1}\right]\right)\left(1+\left(F_{1}\cdot \left[x_{3}\right]\pm F_{2}\cdot \left[x_{d}\right]\right)\right)}$$ (13)

$$v_{2}=\frac{K_{2}\cdot \left[E_{2}\right]\cdot \left[x_{2}\right]}{K_{m_{2}}+\left[x_{2}\right]}$$ (14)

and

$$v_{3}=\frac{K_{3}\cdot \left[E_{3}\right]\cdot \left[x_{3}\right]}{K_{m_{3}}+\left[x_{3}\right]}$$ (15)

Here *K*_1_, *K*_2_ and *K*_3_ are the turnover number/rate constant corresponding to these three reactions. The terms *K*_*m*_1__, *K*_*m*_2__ and *K*_*m*_3__ are Michaelis constants. The metabolite *x*_1_ is generated from the input *I* by a separate reaction catalyzed by an enzyme *E*_0_ having a turnover number *K*_0_ and Michaelis constant *K*_*m*_0__.

Here we include a new term $\frac{1}{1+(\left[F_{1}\cdot x_{3}\right]\pm F_{2}\cdot \left[x_{d}\right])}$ in Eq (13) to incorporate feedback inhibition and various disturbances/perturbations. It indicates that the reaction flux *v*_1_ decreases in proportion to the accumulation of *x*_3_ due to feedback inhibition mechanism on the enzyme *E*_1_, and perturbation *x*_*d*_ (‘+’ indicates that *x*_*d*_ inhibits the reaction and ‘−’ indicates it activates the flux *v*_1_). *F*_1_ and *F*_2_ represent the relative inhibition or activation strength of *x*_3_ and *x*_*d*_ respectively, on the enzyme *E*_1_. The values of feedback constants *F*_1_ and *F*_2_ are chosen randomly in [0, 1]. Similarly, we have defined each reaction flux for both of the original (47 reactions) and reduced (28 reactions) CCM pathway as depicted in S4 and S5 Tables respectively.

Different choices of *F*_1_ and *F*_2_ values just shift the steady state values of different metabolites (states) and reaction fluxes (outputs), as depicted in S2 and S3 Figs. Here we have considered that *x*_*d*_ inhibits the reaction flux *v*_1_. Moreover, we have taken fixed values (0.1) of *F*_2_ and *F*_1_ (S2 and S3 Figs). Subsequently, *F*_1_ and *F*_2_ have been varied in [0, 1] as shown in S2 and S3 Figs respectively. Consequently, in the case of S2 Fig, it can be noticed that as long as the value of *F*_1_ remains at 0.05, the steady state values of *x*_1_, *x*_2_, *x*_3_, *v*_1_, *v*_2_ and *v*_3_ are 0.058, 0.2059, 0.1922, 0.2604, 0.2603 and 0.2604 respectively. When *F*_1_ value increases, reaction flux values of *v*_1_, *v*_2_, *v*_3_ decrease due to strong inhibition activity of *x*_3_ on the enzyme *E*_1_. As a result, the production of *x*_2_ and *x*_3_ decreases. However, the concentration of *x*_1_ increases due to continuous supply of *x*_1_ from the input *I* by a separate reaction as mentioned earlier. In this fashion, *x*_1_ is accumulated because of its less consumption through the reaction *v*_1_. When *F*_1_ value remains at 0.97, *x*_2_, *x*_3_, *v*_1_, *v*_2_ and *v*_3_ reach the steady state with values 0.1924, 0.1794, 0.2529, 0.2530 and 0.2530 respectively. Again, on decreasing *F*_1_ value, the reverse phenomenon can be observed. Similar behaviour can be seen in S3 Fig for fixed *F*_1_ at 0.1 and varying *F*_2_. Thus, it can be concluded from these studies that values of feedback constants (*F*_1_ and *F*_2_) do not affect the pattern of the dynamic responses of a biological system (metabolic pathway). Different choices of feedback constant values only change (increase/decrease) the steady state values. Moreover, the responses of the system always reach the steady states. The results never show any oscillating or exponential nature. Thus, the proposed model is quite robust with respect to the choice of feedback constant value.

Finally, the state space model is obtained by using Eqs (8), (11) and (12), and is given by

$$[\begin{array}{c} \dot{x_{1}}\\ \dot{x_{2}}\\ \dot{x_{3}} \end{array}]=[\begin{array}{ccc} -1 & 0 & 0\\ 1 & -1 & 0\\ 0 & 1 & -1 \end{array}]\cdot [\begin{array}{c} v_{1}\\ v_{2}\\ v_{3} \end{array}]$$ (16)

and

$$y=[\begin{array}{c} v_{1}\\ v_{2}\\ v_{3} \end{array}]$$ (17)

Thus we have got the time (*t*) derivative of state vector (${\left[\dot{x_{1}}\;\dot{x_{2}}\;\dot{x_{3}}\right]}^{T}$), *i.e.*, concentrations of metabolites employed in the hypothetical pathway, and the output *y*, *i.e.*, the flux of the reactions in the pathway using the aforesaid methodology. This is done by solving Eqs (16) and (17) using matlab code.

**Fig 6:**
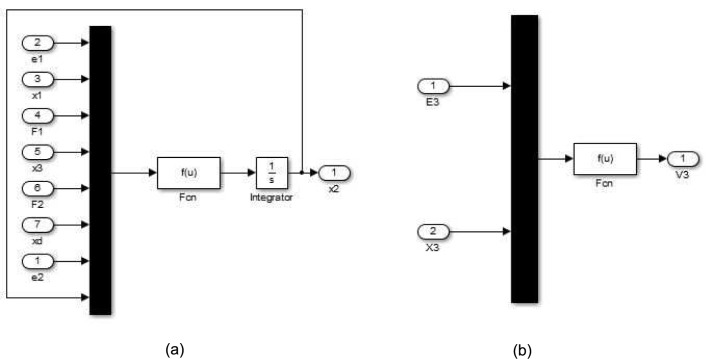
Simulink model (inner layer). It depicts (a) the inner most layer of the proposed model determines a state (*x*_2_) of the hypothetical metabolic pathway in Fig 5 and (b) the inner most layer of the proposed model determines a output (*v*_3_) of the hypothetical metabolic pathway in Fig 5.

**Fig 7:**
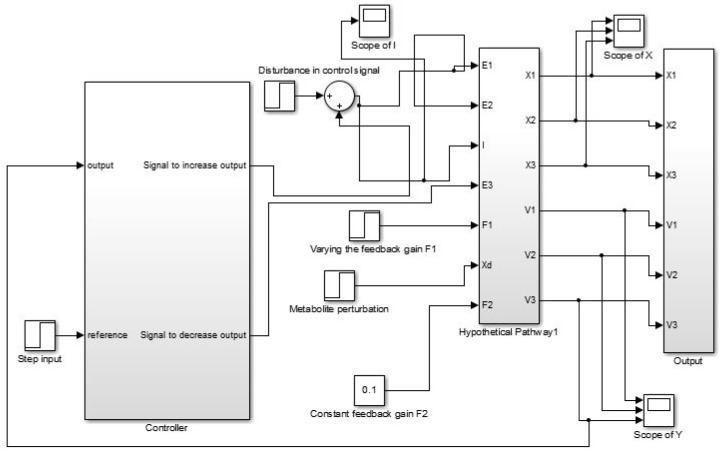
Simulink model (outer layer). It is the outer layer of the proposed model for the hypothetical metabolic pathway in Fig 5.

We have modeled the hypothetical pathway using the control system tool box in the platform of simulink of matlab. We have put the time (*t*) derivative of state vector and output vector in the appropriate function blocks (Fig 6a and 6b) of the model. Fig 7 depicts the outer layer of the proposed model (the hypothetical metabolic pathway in Fig 5). There are several layers of subsystems inside it. Fig 6(a) shows an inner most layer/module of the model, *i.e.*, nothing but a state space model, which determines a state (concentration of a metabolite (here it is *x*_2_ of the hypothetical metabolic pathway in Fig 5)) of the pathway. Besides, there is another kind of inner most layer of the model to determine an output (here it is *v*_3_ for the hypothetical metabolic pathway in Fig 5) of the pathway as shown in Fig 6(b).

The subsystem block, named hypothetical pathway (Fig 7), represents the metabolic pathway, which generates output $e_{i}^{O}$ on application of different inputs $e_{i}^{E}$ and $e_{i}^{I}$. The input is applied to the metabolic pathway using either step input block/signal builder block or signal generated by the controller subsystem block. Mux block (Fig 6a and 6b) is used in the inner most layer to combine inputs/parameters, considered as scaler or vector signal of the same numeric data type, of the pathway into a single output vector. On the other hand, the integrator block generates the value of the integral of its input signal with respect to time. The scope block displays signal(s)/output(s) generated by metabolic pathway with respect to simulation time.

**Fig 8:**
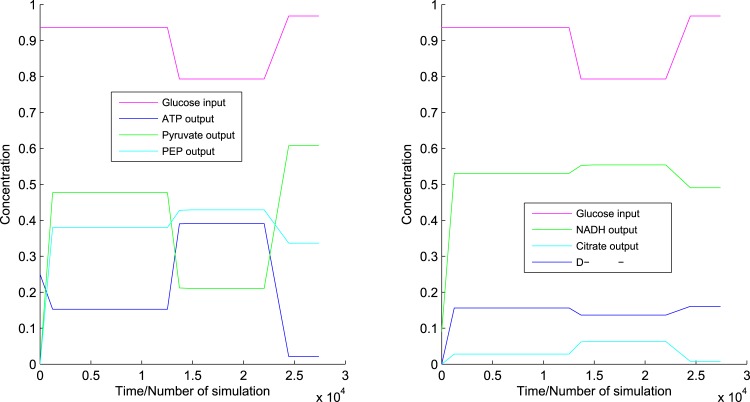
Responses to glucose. It shows the dynamics of CCM pathway in normal cells with respect to glucose.

**Fig 9:**
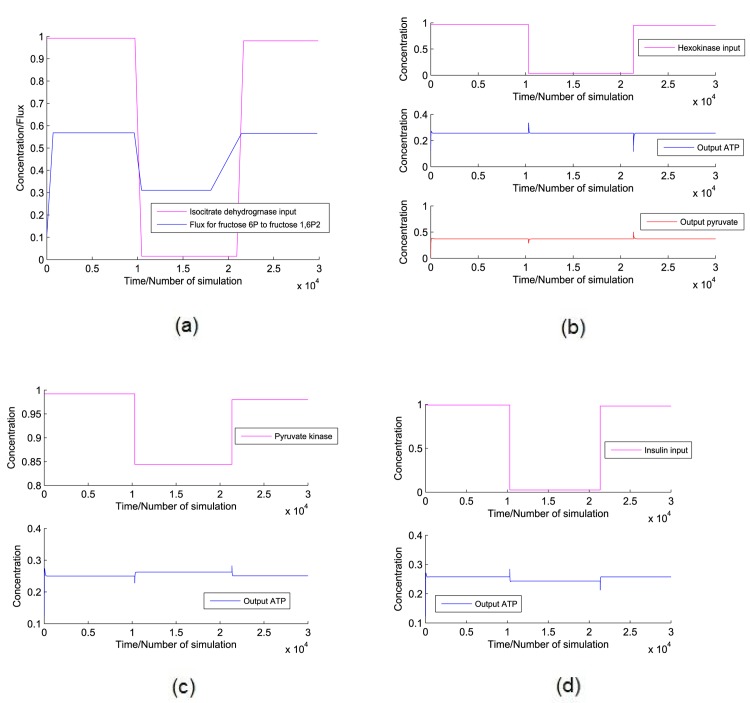
Responses to other perturbations. It captures the dynamics of CCM pathway in normal cells with respect to (a) isocitrate dehydrogenase, (b) hexokinase, (c) pyruvate kinase and (d) insulin.

We have developed the model that mimics the actual dynamics of CCM pathway in the normal cells by applying the aforesaid methodology. The different steps are summarized below:

- Develop the stoichiometric matrix (**N**_*CCM*_) for the reactions depicted in S3 Table.
- Define reaction fluxes of the CCM pathway with modified Michaelis-Menten kinetic equations as depicted in S5 Table.
- Use Eqs (11) and (12) for the CCM pathway, and run the matlab code to generate the time (*t*) derivative of state (metabolite) and output (reaction flux) vectors for the CCM pathway.
- For the state vector:
  – Develop subsystems for each of the states (metabolites) belonging to the CCM pathway using simulink platform of matlab. Each subsystem consists of the components as shown in Fig 6(a).
  – Put the expression for the time (*t*) derivative of state (metabolite) into the function (Fcn) block of individual subsystem.
  – Replace the variables in the expression by u(1), u(2), u(3), …
  – Set the number of inputs (same as the number of variables in the expression of time (*t*) derivative of state) for the mux block.
  – Connect correct inputs to mux block and all the components of the subsystem accordingly.
  – Connect the correct output representing the state (metabolite) to the integrator block.
  – Finally, connect all such subsystems to build a super subsystem representing the state (metabolite) vector.
- Follow the above steps to build a super subsystem (each subsystem being similar to Fig 6(b)) representing the output (reaction flux) vector.
- Connect state vector system and output vector system to develop the proposed model similar to Fig 7, excluding the controller subsystem.
- Supply initial input values for $e_{i}^{E}$, $e_{i}^{I}$, kinetic parameters (rate constants and Michaelis Menten constants) and feedback constants (*F*_1_, *F*_2_, *F*_3_, …). Choose initial input values for $e_{i}^{E}$ in [0, 1]. (A value nearly equal to zero indicates that the gene coding for the respective enzyme has low expression value. On the other hand, the high expression of the gene corresponds to the value being nearly equal to 1.).
- Vary the input values one by one (keeping others at constant values) by step input block/signal builder block. Connect scope block to the varying input to track it.
- Connect scope blocks to each output state (metabolite) and reaction flux to track the changes in responses of the CCM pathway.
- Configure the simulation parameters as described in S6 Table. Run the simulation. Normalize the outputs in [0, 1]. Analyze the results in response to each perturbation (as shown in Figs 8 and 9) and validate.

### Model of the CCM pathway mimicing the altered dynamics in cancer cells

Here we have perturbed the normal model and connect PI controllers with it. Thereafter, we have tuned PI controller to mimic the altered dynamics of mammalian CCM pathway in cancer cells. The basic steps to develop the CCM cancer model from normal CCM model are as follows.

#### Perturbing the normal model

Let $e_{i}^{E}$ and $e_{j}^{E}$ be the concentration of enzymes pyruvate dehydrogenase and pyruvate carboxylase respectively. We have set $e_{i}^{E}$ and $e_{j}^{E}$ to nearly 0 so that the reactions pyruvate → acetyl-coA (reaction number (8) in S3 Table) and pyruvate → oxaloacetate (reaction number (9) in S3 Table) get slowed down. Thus, the proposed model reduces mitochondrial oxidative phosphorylation as in the case of cancer cells.

#### Connecting PI controller(s) and tuning it

As cancer cells show high proliferation, they need sufficient amount of energy in the form of ATP to survive. Therefore, we have connected two PI controllers to the model (as similar as shown in Fig 7) that produces sufficient amount of ATP and cell building materials, *i.e.*, D-ribose-5P or PEP. Let $e_{i}^{D}$, $e_{j}^{D}$ and $e_{k}^{D}$ represent reference/desired concentrations of ATP, D-ribose-5P and PEP respectively. Subsequently, let $e_{i}^{O}$, $e_{j}^{O}$ and $e_{k}^{O}$ be the resultant/actual concentrations of ATP, D-ribose-5P and PEP respectively. One of the two PI controllers try to minimize $|e_{i}^{D}-e_{i}^{O}|$ while the other tries to minimize $|e_{j}^{D}-e_{j}^{O}|$ or $|e_{k}^{D}-e_{k}^{O}|$. However, we have stabilized the model experimentally to mimic the altered dynamics of CCM pathway in cancer cells. Thus, we have considered two cases as follows.

**Fig 10:**
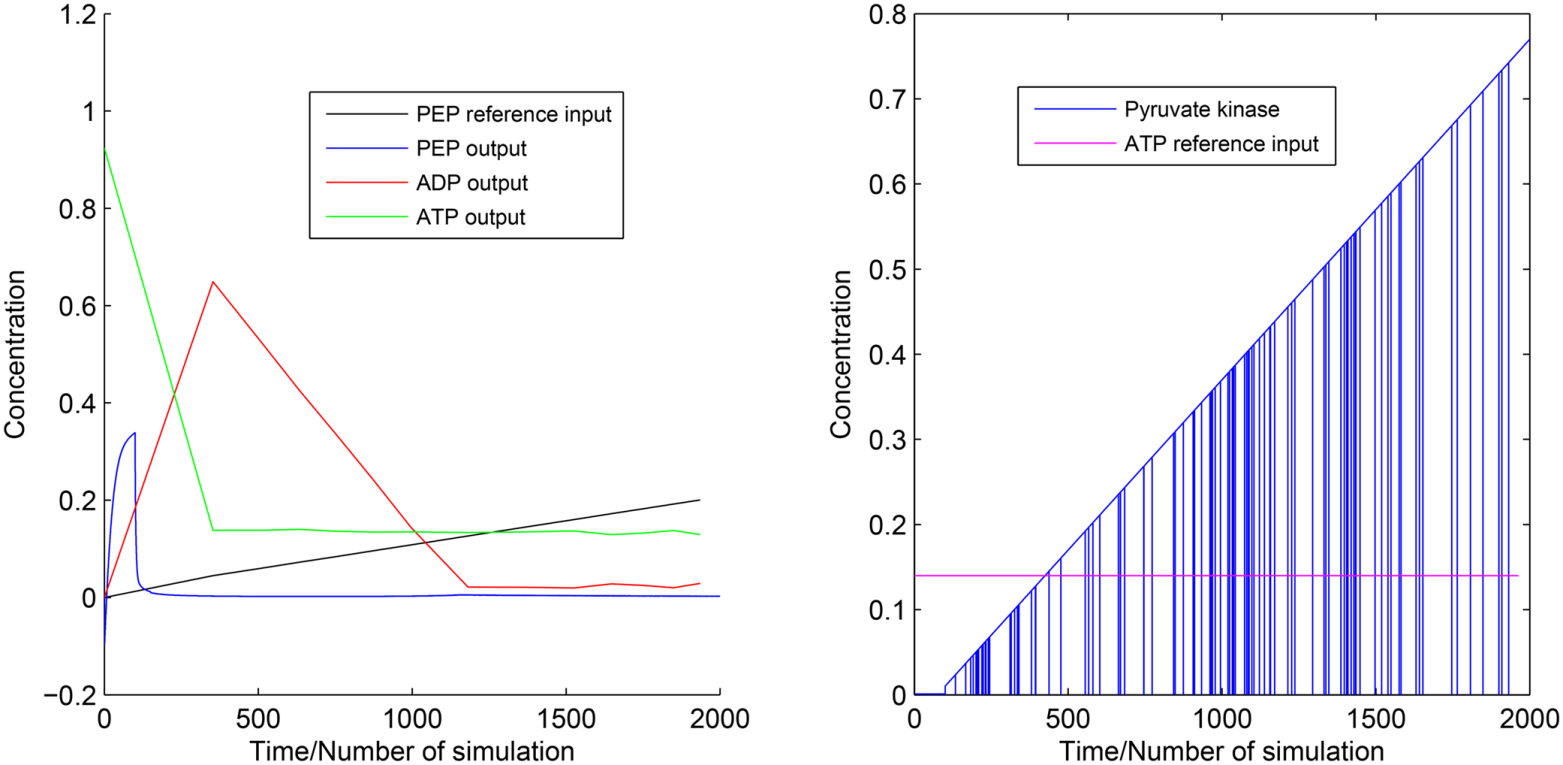
Experiment 1. It shows reference inputs for PEP and ATP along with outputs for PEP, ATP and ADP. It also depicts the switching of pyruvate kinase in [0, 1].

**Fig 11:**
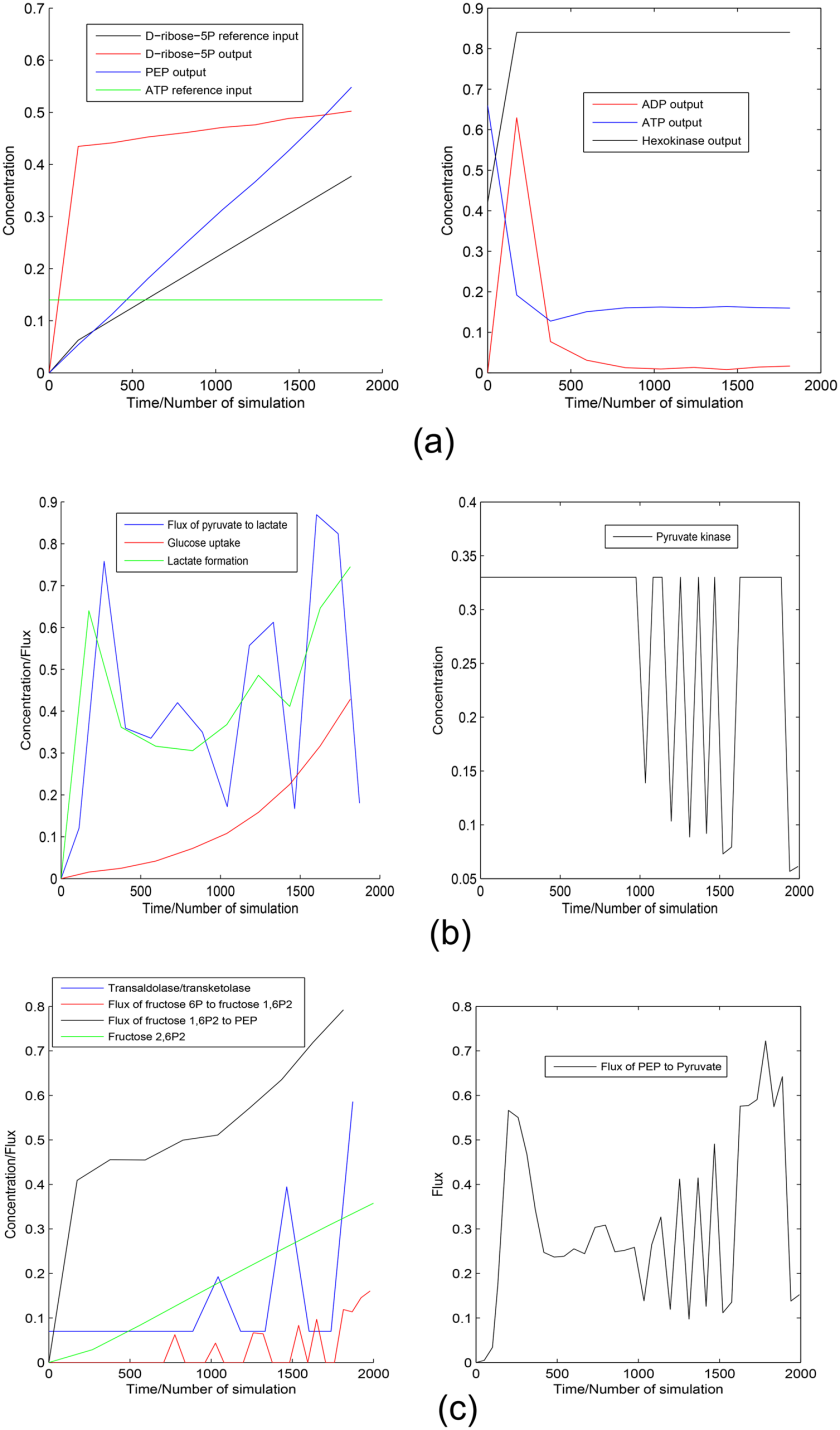
Experiment 2. It depicts (a) the reference inputs for D-ribose-5P and ATP along with the outputs for PEP, ADP, D-ribose-5P, ATP and hexokinase, (b) the flux of pyruvate → lactate, glucose uptake and lactate formation along with the switching of pyruvate kinase in [0, 1] and (c) the regulation of transaldolase/transketolase, fluxes of fructose 6P → fructose 1, 6*P*_2_ and fructose 1, 6*P*_2_ → PEP as well as accumulation of fructose 2, 6*P*_2_. It also shows the fluctuation of the flux of PEP → pyruvate in accordance with fluctuating pyruvate kinase regulation.

- **Case 1:** Here, we have taken PEP as the main source of cell building material. We have tried to increase PEP monotonically by connecting ramp input reference to the first PI controller. Subsequently, actual output concentration of PEP has been supplied to PI controller. Now, the first PI controller will generate control signals (following the Eq (7)) according to the error function (Eq (6)). The control signals has been connected to the required inputs (responsible for PEP production/consumption) of the proposed normal model of CCM pathway to increase/decrease the PEP production. Similarly, another PI controller is used to control ATP production so that sufficient amount of energy can be supplied for the survival of cancer cell. In this scenario, we have run the simulation to generate the altered dynamics of CCM pathway as shown in Fig 10.
- **Case 2:** Here, we have followed the same approach as in case 1, except D-ribose-5P has been considered as the main source of cell building material. The simulation result can be found in Fig 11.

## Results and Discussions

Here we have discussed the behaviour of CCM pathway in normal cells first. Subsequently, we have explored the key enzyme regulations for mammalian CCM pathway in cancer cells.

### Model of normal CCM pathway

We have validated the model of CCM pathway in normal cells using existing literatures [5, 12, 21, 22]. We have varied the concentration of every possible key metabolites with perturbations/hormones ($e_{i}^{I}$) and enzymes ($e_{i}^{E}$) to observe how it affects the dynamics of CCM pathway in normal cells. However, we have included a few results here to restrict the size of article.

#### Variation of glucose

Glucose is broken down into *α*-D-glucose-6P with the consumption of ATP in the earlier phase of glycolysis. Here we have varied concentrations of glucose in an ad hoc manner. We have noticed that due to decrease in glucose level, ATP production rate increases, and vice versa (Fig 8). The reason behind this phenomenon is that due to decrease in glucose level, ATP consumption rate decreases in the earlier phase of glycolysis but in the later phase ATP production does not get affected. However, the production rate of pyruvate changes according to glucose level in the cell (Fig 8). In the case of PEP, the scenario is different. The reaction PEP → pyruvate, *i.e.*, the enzyme pyruvate kinase, is inhibited by ATP [12]. When ATP production rate increases, the reaction PEP → pyruvate slows down. That is why concentration of PEP increases with decrease in glucose level, and vice versa (Fig 8).

Here we have included the reaction pyruvate → lactate which is used in propanoate metabolism. Nicotinamide adenine dinucleotide (reduced form) (NADH) is consumed in this reaction. As pyruvate and lactate formation rates decrease with the decrease in glucose level, consumption of NADH is low. Thus the rate of formation of NADH increases with decrease in glucose level (Fig 8). Again NADH is the main inhibitor of citrate consumption reactions in TCA cycle [12]. Here we have noticed increase in citrate production rate due to excess NADH (Fig 8). D-ribose-5P is produced in pentose phosphate pathway. The switching between pentose phosphate pathway and glycolysis depends on the concentration of reduced NADPH. If the concentration of reduced NADPH is low, then pentose phosphate pathway is activated [12]. Here we have considered reduced NADPH of constant concentration. Thus, due to variation in glucose level, concentration of D-ribose-5P varies in similar manner as of glucose (Fig 8).

#### Variation of isocitrate dehydrogenase

Isocitrate dehydrogenase enzyme is responsible for transformation of isocitrate to *α*-ketoglutarate. Isocitrate is produced from citrate in a reversible reaction. *α*-ketoglutarate is further transformed into succinyl-CoA. NADH acts only as inhibitor of these entire activities [12]. On the other hand, calcium and adenosine diphosphate (ADP) accelerate the same [12]. It is clear from literature [12] that citrate is an inhibitor of PFK1 that activates the reaction *β*-D-fructose-6P → *β*-D-fructose 1, 6*P*_2_. ATP is again consumed in this reaction. Now we have varied concentration of the isocitrate dehydrogenase. When the concentration of the enzyme decreases, overall reaction flux of citrate → … → succinyl-CoA also decreases. As a result, citrate is accumulated in cells. Citrate inhibits the reaction *β*-D-fructose-6P → *β*-D-fructose 1, 6*P*_2_ [12]. Fig 9(a) depicts the change of flux for the reaction *β*-D-fructose-6P → *β*-D-fructose 1, 6*P*_2_ with respect to the isocitrate dehydrogenase.

#### Variation of hexokinase

Hexokinase is responsible for breaking down of glucose into *α*-D-glucose-6P consuming ATP. Now, if we vary concentration of hexokinase in an ad hoc manner, we can see the change of concentrations of ATP and pyruvate accordingly. At that point, where the concentration of hexokinase decreases sharply, ATP production rate suddenly grows up due to less ATP consumption in the reaction for breaking down glucose. However, excess amount of ATP itself inhibits the ATP production reactions [12]. As a result, the production rate of ATP slows down sharply to reach steady state (Fig 9(b)). Reverse phenomenon can be seen when concentration of hexokinase increases sharply. Pyruvate shows opposite scenario when concentration of hexokinase decreases sharply because of inhibitory activity of excess ATP (Fig 9(b)). However, concentration of pyruvate increases sharply in accordance with increase in hexokinase due to energy demand (Fig 9(b)).

#### Variation of pyruvate kinase

Pyruvate kinase is a key enzyme for ATP production as well as in PEP to pyruvate conversion. ATP concentration changes according to increase and decrease of pyruvate kinase (Fig 9(c)).

#### Variation of insulin

Insulin plays an important role in glucose breakdown. It accelerates the activities of hexokinase and phosphofructokinase 2 (PFK2) enzymes catalyzing the reactions glucose → *α*-D-glucose-6P and *β*-D-fructose-6P → *β*-D-fructose 2, 6*P*_2_ respectively [12]. Again, *β*-D-fructose 2, 6*P*_2_ accelerates the conversion of *β*-D-fructose-6P into *β*-D-fructose 1, 6*P*_2_ with the help of the enzyme PFK1 [12]. ATP is consumed in both the reactions glucose → *α*-D-glucose-6P and *β*-D-fructose-6P → *β*-D-fructose 1, 6*P*_2_. In this case, if insulin concentration falls sharply, we see sudden spike in ATP concentration due to the aforesaid reasons (Fig 9(d)). Afterwards, ATP shows opposite scenario in accordance with sharp increase in insulin due to the same reason (Fig 9(d)).

### Cancer model

Here we have analyzed the altered dynamics of CCM pathway in cancer cells with the help of two experimental simulations, *viz.*, Experiment 1 and Experiment 2. In this fashion, we have tried to explore how cancer cells increase their proliferation rate and manage the energy crisis in spite of reduced oxidative phosphorylation.

#### Experiment 1

An earlier experiment [23] demonstrates that PEP plays a crucial role in the phosphorylation of the glycolytic enzyme phosphoglycerate mutase (PGAM1). Again, PGAM1 may provide an alternate glycolytic pathway that accelerates the production of ATP. It also allows high glycolytic flux to meet the anabolic metabolism of many proliferating cells. Keeping this scenario of cancer cells in mind, here we have tried to control the concentration of PEP and ATP using two PI controllers. In this fashion, we have tried to meet two major conditions of cancer cells. One will meet high cell proliferation rate. On the other hand, second one will try to supply sufficient amount of ATP in energy crisis in spite of reduced oxidative phosphorylation. In this scenario, PI controllers try to increase energy production phase of glycolysis.

Fig 10 illustrates the simulation results of Experiment 1. Here we can see that the output ATP production rate meets the reference ATP input applied to the PI controller. Therefore, ADP shows opposite behavior of ATP. Pyruvate kinase shows variation between low and high values in [0, 1]. Low value indicates low active pyruvate kinase. On the other hand, high value indicates its high activity. We have found from literature that pyruvate kinase has two isoform, *i.e.*, M1 and M2 [24, 25]. Among these two isoform, M2 (PKM2) is dominant in cancer cells [24–27]. Again, PKM2 is allosterically regulated between its two oligomeric forms, *i.e.*, a less active dimer and more active tetramer, in tumor cells [28–32]. Thus, Fig 10 depicts similar regulation of pyruvate kinase. However, output concentration of PEP does not meet the reference PEP input applied to PI controller. Here, less active pyruvate kinase produces less pyruvate. It enables accumulation of upstream glycolytic metabolites. They help in shifting metabolic fluxes towards pentose phosphate pathway.

Thus, it is possible to produce sufficient amount of D-ribose-5P and reduced NADPH. They help in synthesizing amino acids, nucleotides and lipids (cell building materials) in high proliferating cancer cells [2, 32]. Therefore, the metabolic fluxes through pentose phosphate pathway may perform a major role to maintain altered dynamics of CCM pathway in cancer cells. Furthermore, Experiment 2 has tried to establish the above mentioned hypothesis.

#### Experiment 2

We consider the same approaches in Experiment 2 to control the concentrations of D-ribose-5P and ATP. Fig 11 demonstrates the results of Experiment 2. D-ribose-5P meets the ramp reference input of itself (Fig 11(a)). Besides, PEP is accumulated in cells (Fig 11(a)). ATP production satisfies its reference input (Fig 11(a)). Subsequently, ADP shows opposite scenario as of ATP (Fig 11(a)). Again, hexokinase is over expressed (Fig 11(a)). Fig 11(b) illustrates increase in glucose uptake and lactate production in cancer cells. Besides, flux of the reaction from pyruvate to lactate and concentration of pyruvate kinase are fluctuating between a high and low value in [0, 1]. In this scenario, transaldolase/transketolase shows opposite fluctuation as of pyruvate kinase (Fig 11(c)). Consequently, flux of the reaction from *β*-D-fructose-6P to *β*-D-fructose-1, 6*P*_2_ fluctuates in accordance with transaldolase/transketolase (Fig 11(c)). Higher production rate of *β*-D-fructose-2, 6*P*_2_ (Fig 11(c)) helps to catalyze the reaction *β*-D-fructose-6P → *β*-D-fructose-1, 6*P*_2_. Thus, flux of the reaction *β*-D-fructose-1, 6*P*_2_ → PEP increases rapidly (Fig 11(c)). Subsequently, flux of the reaction from PEP to pyruvate, *i.e.*, ATP formation is fluctuating in accordance with pyruvate kinase (Fig 11(c)).

There are different growth factors in cells that prevent uncontrolled proliferation by limiting unnecessary nutrient uptake. High proliferation of cancer cells overcomes these growth factors by altering different signaling pathways [2]. As a result, glucose uptake increases with over expression of hexokinase [15]. It validates our simulation result. This experiment allows cancer cells to satisfy two major scenarios—one forming cell building materials, other producing sufficient energy. Here ramp output of D-ribose-5P helps in forming nucleotides of cancer cells. PEP accumulation accelerates anabolic metabolism of high proliferating cells with the help of PGAM1. Besides, ATP is formed as per need of the cancer cells during energy crisis.

Previous investigations [24–32] have found that dominant PKM2 is expressed in its more active oligomeric form tetramer when energy is needed. Afterwards, tetramer is transformed into less active dimer when cell building materials are formed. Pentose phosphate pathway is the major supplier of cell building materials. Our simulation result shows similar kind of fluctuation for pyruvate kinase. Another interesting fluctuation is shown by transaldolase/transketolase. When activity of pyruvate kinase slows down, transaldolase/transketolase accelerates the conversion of D-ribose-5P to intermediate glycolytic metabolites. Consequently, these accumulated intermediate glycolytic metabolites are converted back to *α*-D-glucose-6P. Activity of glucose-6-phosphate dehydrogenase converts *α*-D-glucose-6P into intermediate metabolites of pentose phosphate pathway. In this fashion, fluxes of the reactions through pentose phosphate pathway increase to form more D-ribose-5P. Similar kind of results can be found in [1, 2].

Again, in case of tetramer expression of PKM2, these accumulated glycolytic materials (*i.e.*, PEP) accelerate the energy forming phase of glycolysis. We have already discussed that PEP helps in the phosphorylation of PGAM1. It accelerates ATP formation by an alternate glycolytic pathway [23]. Warburg has demonstrated the fermentation of glucose to produce lactate in cancer cells [2, 32]. This finding validates the overproduction of lactate shown by our simulation result. Moreover, it has been observed that in some cancer cells PFK2 activates PFK1 by generating *β*-D-fructose-2, 6*P*_2_ [33]. As a result, fluxes through glycolysis increase. Thus higher rate of *β*-D-fructose-2, 6*P*_2_ formation shown by the simulation result is validated. Consequently, flux of the conversion of *β*-D-fructose-1, 6*P*_2_ into PEP increases rapidly. However, there is a fluctuation in the flux of the reaction *β*-D-fructose-6P → *β*-D-fructose-1, 6*P*_2_ because of the fluctuation in transaldolase/transketolase.

It is clear from the aforesaid observations that switching of pyruvate kinase (M2 isoform) between its two oligomeric forms, tetramer and dimer, plays a major role to maintain the altered dynamics in cancer cells. In addition, transaldolase/transketolase performs a significant role. Hexokinase, glucose-6-phosphate dehydrogenase, PFK2, PFK1 are also significant in this scenario. Thus we can conclude that coordination among enzymes catalyzing glycolysis and pentose phosphate pathway drive cancer cells to fulfill its two important characteristics (*i.e.*, energy formation and high cell proliferation). In this fashion, breaking this kind of coordination can be set as an important therapeutic goal.

## Conclusions

Here we have developed a model to mimic the nonlinear mammalian CCM pathway using classical control theory. The focus of our approach is to explore how the control mechanism works using enzyme regulations to achieve certain objectives of mammalian CCM pathway. We have considered all the control points of the CCM pathway and other interactions.

This modeling technique allows one to perturb each and every parameter associated with the pathway. It also helps in observing the transient and steady state responses. Moreover, the model can track dynamic closed loop responses. Besides, it is also capable of tracking even a ramp input/output which is frequent in cancer cells. Thus, the model becomes more robust than the previous models based on flux balance analysis, metabolic control analysis and classical control theory. The present approach allows one to analyze the possible control action of a pathway that may be followed to attain a specific objective by varying the key enzymes regulating the control points. In this case, we have analyzed the effects of regulation of enzymes in every possible combinations. Besides, we have analyzed the role of perturbations in attaining specific objectives. Through this study we have been able to capture the effects of perturbations of key enzymes regulating the CCM pathway in normal cells. We have also simulated the effects of various metabolites that create disturbances due to feedback and cross talk with interlinking pathways.

As cancer cells alter the normal behavior of the CCM pathway to fulfill high cell proliferation and energy demand, PI controllers have been applied to the model of the normal CCM pathway which has been perturbed to reduce the mitochondrial oxidative phosphorylation. In this fashion, the model behaves like the CCM pathway in mammalian cancer cells. We have analyzed the possible enzyme regulations in cancer cells. In this regard, we have found some interesting results.

Both Experiment 1 and Experiment 2 show fluctuation for the concentration of pyruvate concentration between [0, 1]. According to some previous investigations [2, 14, 24, 25, 27–30, 33–35], pyruvate kinase has two isoforms, *i.e.* M1 and M2. PKM2 is dominant in cancer cells. It has two oligomeric forms (dimer and tetramer). The switching between dimer and tetramer helps cancer cells in fulfilling energy demand and high cell proliferation. Thus, the fluctuation of pyruvate kinase is quite valid. Experiment 1 does not consider the interaction among the enzymes associated with glycolysis and pentose phosphate pathways. Subsequently, Experiment 1 shows that sufficient amount of energy in the form of ATP can not be produced as per need of cancer cells.

Besides, Experiment 2 confirms that there is a switching among the enzymes catalyzing pentose phosphate pathway and intermediate glycolytic enzymes. In addition to this, the switching of PKM2 plays an important role for survival of cancer cells. Fluctuation of transaldolase/transketolase between low and high concentration plays an important role in this scenario. When energy is needed, D-ribose-5P is converted into intermediate glycolytic metabolites, switching PKM2 to its active tetramer form. As a result, desired amount of ATP is synthesized. Production of D-ribose-5P becomes high again during cell proliferation. In this scenario, PKM2 switches itself back to relative inactive dimer form. As a result, PEP gets accumulated (shown by Experiment 2). Afterwards, accumulated PEP helps in forming cell building materials and ATP as per need. Experiment 2 also indicates that the enhanced activities of hexokinase, PFK1, PFK2 and glucose-6-phosphate dehydrogenase contribute significantly to maintain the altered dynamics in cancer cells. Thus, the model can meet the needs of cancer cells (energy demand and high cell proliferation) efficiently. Moreover, it helps cancer cells in surviving in an initial low concentration of ATP. In this fashion, the model successfully mimic the altered dynamics of the mammalian CCM pathway in cancer cells.

## Supporting Information

S1 Fig Comparison between the proposed models of the CCM pathways consisting of 47 reactions and 28 reactions. This two models show the similar pattern of dynamic responses of CCM pathway with variations of glucose and pyruvate kinase.
(TIF)

S2 Fig Significance of the feedback constant (case 1). It shows the variations of *x*_1_, *x*_2_, *x*_3_, *v*_1_, *v*_2_ and *v*_3_ (as depicted in Fig 5) with variation of *F*_1_ value and fixed *F*_2_ value at 0.1.
(TIF)

S3 Fig Significance of the feedback constant (case 2). It depicts the variations of *x*_1_, *x*_2_, *x*_3_, *v*_1_, *v*_2_ and *v*_3_ (as depicted in Fig 5) with variation of *F*_2_ value and fixed *F*_1_ value at 0.1.
(TIF)

S1 Table Simulation details for Fig 3. It contains the simulation details and the model parameters which have been used to validate the order reduction technique using COPASI software.
(PDF)

S2 Table Reaction list 1. It contains the list of 47 reactions from KEGG database for mammalian CCM pathway under consideration. Here the reaction numbers correspond to the same serial numbers in S4 Table.
(PDF)

S3 Table Reaction list 2. It contains the list of reduced 28 reactions for mammalian CCM pathway under consideration. Here the reaction numbers correspond to the same serial numbers in S5 Table.
(PDF)

S4 Table Reaction Eq (1). It contains the list of equations of reaction fluxes associated with 47 reactions from KEGG database for mammalian CCM pathway under consideration. Here the serial numbers correspond to the same reaction numbers in S2 Table.
(PDF)

S5 Table Reaction Eq (2). It contains the list of equations of reaction fluxes associated with reduced 28 reactions from KEGG database for mammalian CCM pathway under consideration. Here the serial numbers correspond to the same reaction numbers in S3 Table.
(PDF)

S6 Table Simulation parameters. Here, the simulation parameters for the proposed CCM pathway model are given.
(PDF)

## References

[1] Resendis-Antonio O, Checa A, Encarnación S. Modeling core metabolism in cancer cells: surveying the topology underlying the Warburg effect. PLoS One. 2010;5(8):e12383 10.1371/journal.pone.0012383 20811631 PMC2928278

[2] Vander Heiden MG, Cantley LC, Thompson CB. Understanding the Warburg effect: the metabolic requirements of cell proliferation. Science. 2009;324(5930):1029–1033. 10.1126/science.1160809 19460998 PMC2849637

[3] Kauffman KJ, Prakash P, Edwards JS. Advances in flux balance analysis. Current Opinion in Biotechnology. 2003;14(5):491–496. 10.1016/j.copbio.2003.08.001 14580578

[4] De RK, Das M, Mukhopadhyay S. Incorporation of enzyme concentrations into FBA and identification of optimal metabolic pathways. BMC Systems Biology. 2008;2(1):65 10.1186/1752-0509-2-65 18634554 PMC2533768

[5] De RK, Tomar N. Modeling the optimal carbon metabolic pathways under feedback inhibition using flux balance analysis. Journal of Bioinformatics and Computational Biology. 2012;10(06). 10.1142/S0219720012500199 22913632

[6] Acerenza L, Sauro HM, Kacser H. Control analysis of time-dependent metabolic systems. Journal of Theoretical Biology. 1989;137(4):423–444. 10.1016/S0022-5193(89)80038-4 2626059

[7] Cascante M, Boros LG, Comin-Anduix B, de Atauri P, Centelles JJ, Lee PWN. Metabolic control analysis in drug discovery and disease. Nature Biotechnology. 2002;20(3):243–249. 10.1038/nbt0302-243 11875424

[8] Kholodenko BN, Demin OV, Westerhoff HV. Control analysis of periodic phenomena in biological systems. The Journal of Physical Chemistry B. 1997;101(11):2070–2081. 10.1021/jp962336u

[9] Wildermuth MC. Metabolic control analysis: biological applications and insights. Genome Biology. 2000;1(6):1031–1. 10.1186/gb-2000-1-6-reviews1031 PMC138895 11178271

[10] Rao, CV, Sauro, HM, Arkin, AP. Putting the control in metabolic control analysis,. In: 7th International Symposium on Dynamics and Control of Process Systems, DYCOPS. vol. 7; 2004.

[11] Panja S, Patra S, Mukherjee A, Basu M, Sengupta S, Dutta PK. A closed-loop control scheme for steering steady states of glycolysis and glycogenolysis pathway. IEEE/ACM Transactions on Computational Biology and Bioinformatics (TCBB). 2013;10(4):858–868. 10.1109/TCBB.2013.82 24334381

[12] Nelson DL, Lehninger AL, Cox MM. Lehninger Principles of Biochemistry. Macmillan; 2008.

[13] Morris KA. Introduction to Feedback Control. Academic Press, Inc.; 2000.

[14] Kroemer G, Pouyssegur J. Tumor cell metabolism: cancer’s Achilles’ heel. Cancer Cell. 2008;13(6):472–482. 10.1016/j.ccr.2008.05.005 18538731

[15] Marín-Hernández A, Gallardo-Pérez JC, Rodríguez-Enríquez S, Encalada R, Moreno-Sánchez R, Saavedra E. Modeling cancer glycolysis. Biochimica et Biophysica Acta (BBA)-Bioenergetics. 2011;1807(6):755–767. 10.1016/j.bbabio.2010.11.006 21110941

[16] Chang R. Physical Chemistry for the Biosciences. University Science Books; 2005.

[17] Gerdtzen ZP, Daoutidis P, Hu WS. Non-linear reduction for kinetic models of metabolic reaction networks. Metabolic Engineering. 2004;6(2):140–154. 10.1016/j.ymben.2003.11.003 15113567

[18] Famili I, Palsson BO. The convex basis of the left null space of the stoichiometric matrix leads to the definition of metabolically meaningful pools. Biophysical Journal. 2003;85(1):16–26. 10.1016/S0006-3495(03)74450-6 12829460 PMC1303061

[19] Qian H, Beard DA, Liang Sd. Stoichiometric network theory for nonequilibrium biochemical systems. European Journal of Biochemistry. 2003;270(3):415–421. 10.1046/j.1432-1033.2003.03357.x 12542691

[20] Hoops S, Sahle S, Gauges R, Lee C, Pahle J, Simus N, et al COPASIa complex pathway simulator. Bioinformatics. 2006;22(24):3067–3074. 10.1093/bioinformatics/btl485 17032683

[21] Chassagnole C, Noisommit-Rizzi N, Schmid JW, Mauch K, Reuss M. Dynamic modeling of the central carbon metabolism of Escherichia coli. Biotechnology and Bioengineering. 2002;79(1):53–73. 10.1002/bit.10288 17590932

[22] Shlomi T, Cabili MN, Herrgård MJ, Palsson BØ, Ruppin E. Network-based prediction of human tissue-specific metabolism. Nature Biotechnology. 2008;26(9):1003–1010. 10.1038/nbt.1487 18711341

[23] Vander Heiden MG, Locasale JW, Swanson KD, Sharfi H, Heffron GJ, Amador-Noguez D, et al Evidence for an alternative glycolytic pathway in rapidly proliferating cells. Science. 2010;329(5998):1492–1499. 10.1126/science.1188015 20847263 PMC3030121

[24] Christofk HR, Vander Heiden MG, Harris MH, Ramanathan A, Gerszten RE, Wei R, et al The M2 splice isoform of pyruvate kinase is important for cancer metabolism and tumour growth. Nature. 2008;452(7184):230–233. 10.1038/nature06734 18337823

[25] Clower CV, Chatterjee D, Wang Z, Cantley LC, Vander Heiden MG, Krainer AR. The alternative splicing repressors hnRNP A1/A2 and PTB influence pyruvate kinase isoform expression and cell metabolism. Proceedings of the National Academy of Sciences, USA. 2010;107(5):1894–1899. 10.1073/pnas.0914845107 PMC2838216 20133837

[26] Huang L, Yu Z, Zhang T, Zhao X, Huang G. HSP40 interacts with pyruvate kinase M2 and regulates glycolysis and cell proliferation in tumor cells. PLoS One. 2014;9(3):e92949 10.1371/journal.pone.0092949 24658033 PMC3962495

[27] Christofk HR, Vander Heiden MG, Wu N, Asara JM, Cantley LC. Pyruvate kinase M2 is a phosphotyrosine-binding protein. Nature. 2008;452(7184):181–186. 10.1038/nature06667 18337815

[28] Anastasiou D, Yu Y, Israelsen WJ, Jiang JK, Boxer MB, Hong BS, et al Pyruvate kinase M2 activators promote tetramer formation and suppress tumorigenesis. Nature Chemical Biology. 2012;8(10):839–847. 10.1038/nchembio.1060 22922757 PMC3711671

[29] Iqbal MA, Gupta V, Gopinath P, Mazurek S, Bamezai RN. Pyruvate kinase M2 and cancer: an updated assessment. Federation of European Biochemical Societies Letters. 2014;. 10.1016/j.febslet.2014.04.011 24747424

[30] Mazurek S. Pyruvate kinase type M2: a key regulator of the metabolic budget system in tumor cells. The International Journal of Biochemistry & Cell Biology. 2011;43(7):969–980. 10.1016/j.biocel.2010.02.005 20156581

[31] Hitosugi T, Kang S, Vander Heiden MG, Chung TW, Elf S, Lythgoe K, et al Tyrosine phosphorylation inhibits PKM2 to promote the Warburg effect and tumor growth. Science Signaling. 2009;2(97):ra73 10.1126/scisignal.2000431 19920251 PMC2812789

[32] Wong N, De Melo J, Tang D. PKM2, a central point of regulation in cancer metabolism. International Journal of Cell Biology. 2013;2013 10.1155/2013/242513 23476652 PMC3586519

[33] Vander Heiden MG. Targeting cancer metabolism: a therapeutic window opens. Nature Reviews Drug Discovery. 2011;10(9):671–684. 10.1038/nrd3504 21878982

[34] Luo W, Semenza GL. Emerging roles of PKM2 in cell metabolism and cancer progression. Trends in Endocrinology & Metabolism. 2012;23(11):560–566. 10.1016/j.tem.2012.06.010 22824010 PMC3466350

[35] Mazurek S. Pyruvate kinase M2: A key enzyme of the tumor metabolome and its medical relevance. Biomed Res. 2012;23:133–141.

