# Supplementary figures and images for: Exploring the Altered Dynamics of Mammalian Central Carbon Metabolic Pathway in Cancer Cells: A Classical Control Theoretic Approach
Source: PLoS One. 2015 Sep 14;10(9):e0137728. doi: 10.1371/journal.pone.0137728 (PMC4569588; doi:10.1371/journal.pone.0137728)

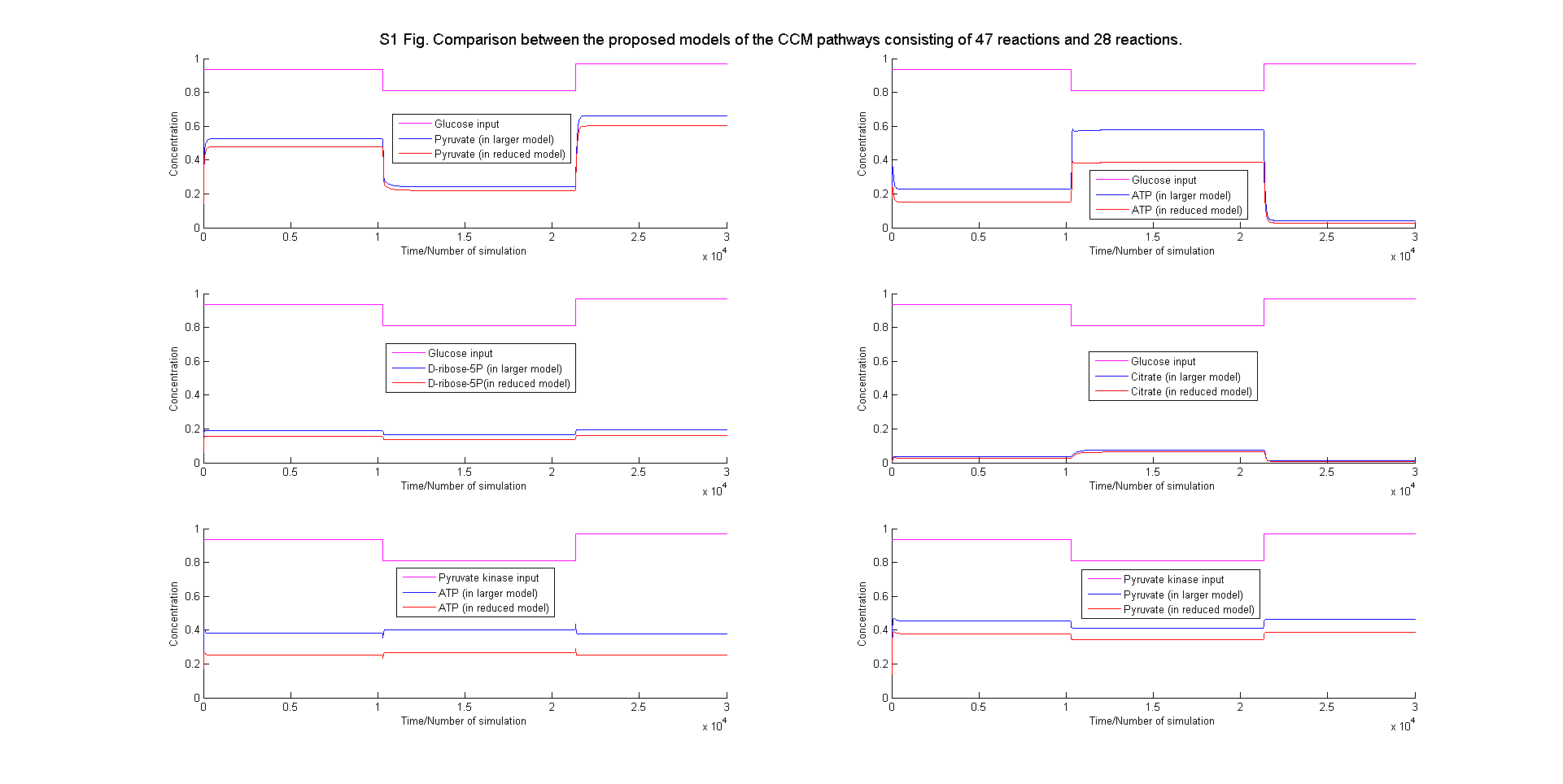

Supplement: S1 Fig — This two models show the similar pattern of dynamic responses of CCM pathway with variations of glucose and pyruvate kinase. (TIF) [file pone.0137728.s001.tif]

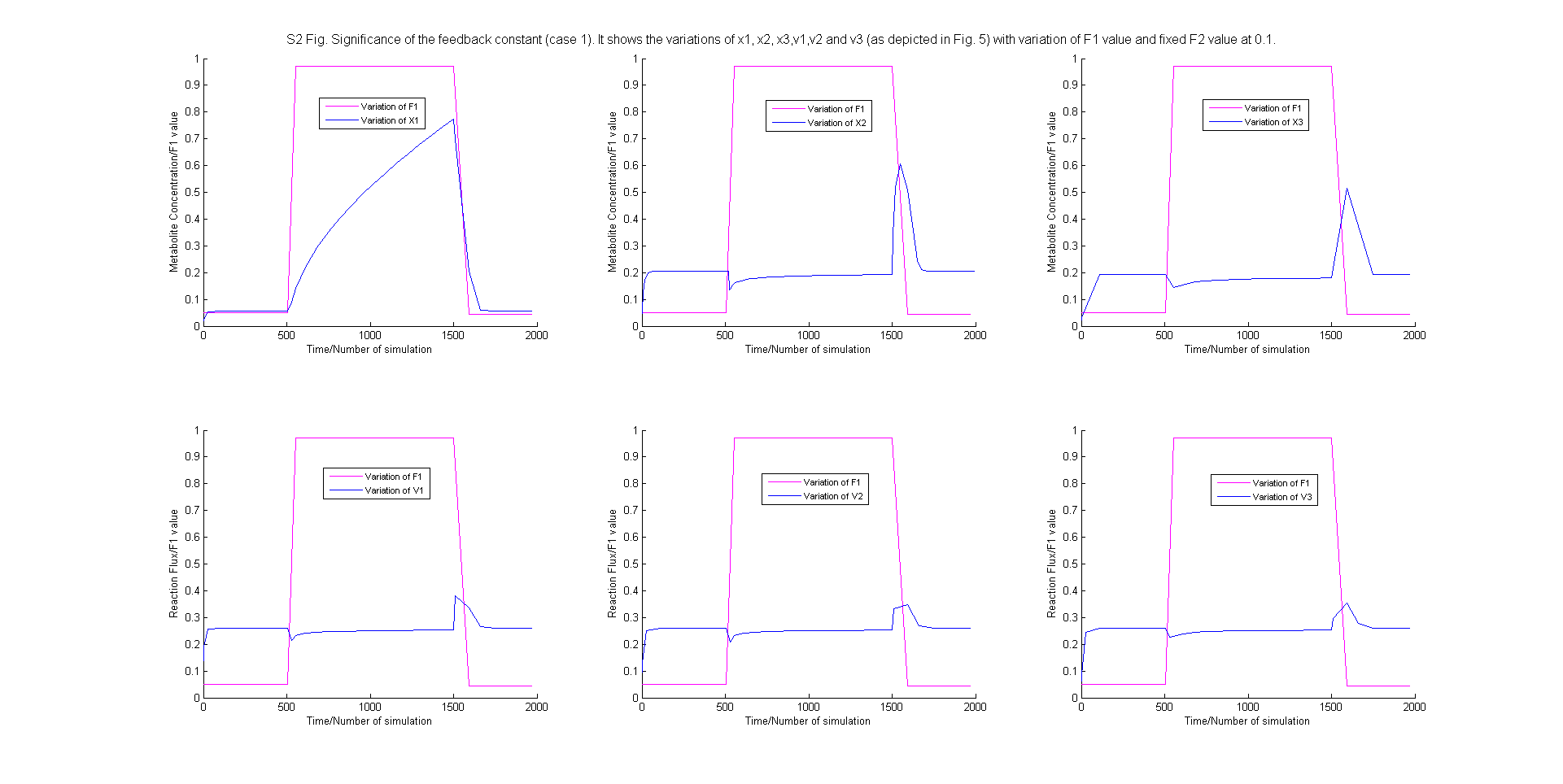

Supplement: S2 Fig — It shows the variations of x 1, x 2, x 3, v 1, v 2 and v 3 (as depicted in Fig 5) with variation of F 1 value and fixed F 2 value at 0.1. (TIF) [file pone.0137728.s002.tif]

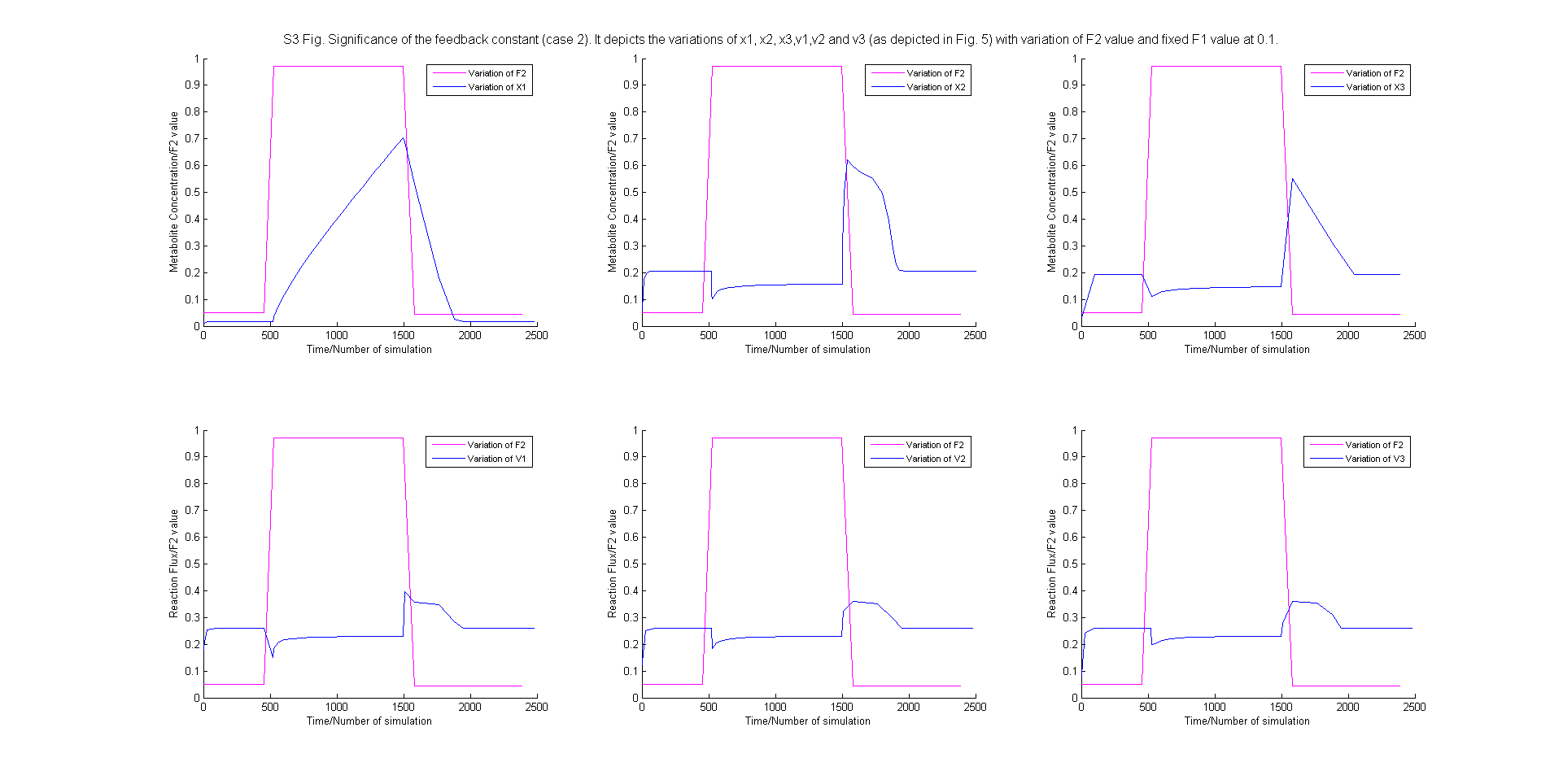

Supplement: S3 Fig — It depicts the variations of x 1, x 2, x 3, v 1, v 2 and v 3 (as depicted in Fig 5) with variation of F 2 value and fixed F 1 value at 0.1. (TIF) [file pone.0137728.s003.tif]
