# Supplementary material for: Exploring the Altered Dynamics of Mammalian Central Carbon Metabolic Pathway in Cancer Cells: A Classical Control Theoretic Approach
Source: PLoS One. 2015 Sep 14;10(9):e0137728. doi: 10.1371/journal.pone.0137728 (PMC4569588; doi:10.1371/journal.pone.0137728)
Supplement: S1 Table — It contains the simulation details and the model parameters which have been used to validate the order reduction technique using COPASI software. (PDF) [file pone.0137728.s004.pdf]

**S1 Table. Simulation details for Fig. 3.** It contains the simulation details and the model parameters which have been used to validate the order reduction technique using COPASI software.

**A: Simulation information**

| Simulation specification | | |
|---|---|---|
| Simulation task | Time course | |
| Duration | 500 s | |
| Interval size | 5 s | |
| Intervals | 100 | |
| Method | Deterministic (LSODA) | |
| Parameter | Integrate reduced model | 0 |
| | Relative tolerance | $1\times10^{-6}$ |
| | Absolute tolerance | $1\times10^{-12}$ |
| | Max internal steps | 10000 |

**B: Model information**

| Original pathway (Fig. 3(a)) model specifications | | |
|---|---|---|
| Initial Species Values | | |
| | A | 0.5 mmol/ml |
| | B | 0.5 mmol/ml |
| | C | 0.5 mmol/ml |
| | D | 0.5 mmol/ml |
| Kinetic Parameters | | |
| $v_1$ (Rate law: Noncompetitive inhibition (irr)) | | |
| | $K_m$ | 0.1 mmol/ml |
| | $V$ | 0.1 mmol/(ml×s) |
| | $K_i$ | 0.1 mmol/ml |
| $v_2$ (Rate law: Henri-Michaelis-Menten (irr)) | | |
| | $K_m$ | 0.1 mmol/ml |
| | $V$ | 0.1 mmol/(ml×s) |
| $v_3$ (Rate law: Henri-Michaelis-Menten (irr)) | | |
| | $K_m$ | 0.1 mmol/ml |
| | $V$ | 0.1 mmol/(ml×s) |
| **Reduced pathway (Fig. 3(b)) model specifications** | | |
| Initial Species Values | | |
| | B | 0.5 mmol/ml |
| | D | 0.5 mmol/ml |
| | A | 0.5 mmol/ml |
| Kinetic Parameters | | |
| $v_1$ (Rate law: Noncompetitive inhibition (irr)) | | |
| | $K_m$ | 0.1 mmol/ml |
| | $V$ | 0.1 mmol/(ml×s) |
| | $K_i$ | 0.1 mmol/ml |
| $v_2$ (Rate law: Henri-Michaelis-Menten (irr)) | | |
| | $K_m$ | 0.1 mmol/ml |
| | $V$ | 0.1 mmol/(ml×s) |
