# Supplementary material for: Exploring the Altered Dynamics of Mammalian Central Carbon Metabolic Pathway in Cancer Cells: A Classical Control Theoretic Approach
Source: PLoS One. 2015 Sep 14;10(9):e0137728. doi: 10.1371/journal.pone.0137728 (PMC4569588; doi:10.1371/journal.pone.0137728)
Supplement: S2 Table — It contains the list of 47 reactions from KEGG database for mammalian CCM pathway under consideration. Here the reaction numbers correspond to the same serial numbers in S4 Table. (PDF) [file pone.0137728.s005.pdf]

S2 Table. Reaction list 1. It contains the list of 47 reactions from KEGG database for mammalian CCM pathway under consideration. Here the reaction numbers correspond to the same serial numbers in S4 Table.

1. Glucose + ATP $\Rightarrow$ α-D glucose 6P + ADP
2. α-D glucose 6P $\Rightarrow$ Glucose
3. β-D glucose 6P $\Rightarrow$ α-D glucose 6P
4. β-D glucose 6P $\Rightarrow$ β-D fructose 6P
5. α-D glucose 6P $\Rightarrow$ β-D fructose 6P
6. β-D fructose 6P + ATP $\Rightarrow$ β-D fructose 1,6P_2 + ADP
7. β-D fructose 6P $\Rightarrow$ β-D fructose 2,6P_2
8. β-D fructose 2,6P_2 $\Rightarrow$ β-D fructose 6P
9. β-D fructose 1,6P_2 $\Rightarrow$ D-Glyceraldehyde 3P + NADH
10. β-D fructose 1,6P_2 $\Rightarrow$ Glycerone P
11. Glycerone P $\Rightarrow$ D-Glyceraldehyde 3P
12. D-Glyceraldehyde 3P $\Rightarrow$ Glycerate 1,3P_2
13. Glycerate 1,3P_2 + ADP $\Rightarrow$ Glycerate 3P + ATP
14. Glycerate 3P $\Rightarrow$ Glycerate 2P
15. Glycerate 2P $\Rightarrow$ Phosphoenolpyruvate (PEP)
16. Phosphoenolpyruvate (PEP) + ADP $\Rightarrow$ Pyruvate + ATP
17. Oxaloacetate $\Rightarrow$ PEP
18. Pyruvate $\Rightarrow$ 2-hydroxy-ethyl-ThPP
19. 2-hydroxy-ethyl-ThPP $\Rightarrow$ S-acetyldihydro-lipoamide-E
20. S-acetyldihydro-lipoamide-E $\Rightarrow$ Acetyl-CoA
21. Pyruvate $\Rightarrow$ Oxaloacetate
22. Oxaloacetate + Acetyl-CoA $\Rightarrow$ Citrate
23. Citrate $\Rightarrow$ Cis Aconitate
24. Cis Aconitate $\Rightarrow$ Isocitrate
25. Isocitrate $\Rightarrow$ Oxalosuccinate
26. Oxalosuccinate $\Rightarrow$ 2-oxoglutarate (α-ketoglutarate) + NADH
27. 2-oxoglutarate $\Rightarrow$ 3-carboxy-1-hydroxypropyl-ThPP
28. 3-carboxy-1-hydroxypropyl-ThPP $\Rightarrow$ S-succinyl-dihydrolipoamide-E
29. S-succinyl-dihydrolipoamide-E $\Rightarrow$ Succinyl-CoA + NADH
30. Succinyl-CoA $\Rightarrow$ Succinate + GTP (ATP)
31. Succinate $\Rightarrow$ Fumarate
32. Fumarate $\Rightarrow$ (S)-malate
33. (S)-malate $\Rightarrow$ Oxaloacetate + NADH
34. α-D glucose 6P $\Rightarrow$ β-D glucose 6P
35. β-D glucose 6P $\Rightarrow$ D-glucono-1,5-lactone-6P
36. D-Glucono-1,5-lactone-6P $\Rightarrow$ 6-phosphogluconate + NADPH
37. 6-phosphogluconate $\Rightarrow$ Ribulose 5-phosphate + NADPH
38. Ribulose 5-phosphate $\Rightarrow$ D-ribose-5P
39. Ribulose 5-phosphate $\Rightarrow$ Xylulose 5-phosphate
40. D-ribose-5P + Xylulose 5-phosphate $\Rightarrow$ D-Glyceraldehyde 3P + Sedoheptulose 7-phosphate
41. D-Glyceraldehyde 3P $\Rightarrow$ β-D fructose 1,6P_2
42. β-D fructose 1,6P_2 $\Rightarrow$ β-D fructose 6P
43. β-D fructose 6P $\Rightarrow$ α-D glucose 6P
44. Sedoheptulose 7-phosphate + D-Glyceraldehyde 3P $\Rightarrow$ Erythrose 4-phosphate + β-D fructose 6P
45. Xylulose 5-phosphate + Erythrose 4-phosphate $\Rightarrow$ β-D fructose 6P + D-Glyceraldehyde 3P
46. Pyruvate + NADH $\Rightarrow$ Lactate
47. Pyruvate $\Rightarrow$ Alanine
