# Supplementary material for: Exploring the Altered Dynamics of Mammalian Central Carbon Metabolic Pathway in Cancer Cells: A Classical Control Theoretic Approach
Source: PLoS One. 2015 Sep 14;10(9):e0137728. doi: 10.1371/journal.pone.0137728 (PMC4569588; doi:10.1371/journal.pone.0137728)
Supplement: S3 Table — It contains the list of reduced 28 reactions for mammalian CCM pathway under consideration. Here the reaction numbers correspond to the same serial numbers in S5 Table. (PDF) [file pone.0137728.s006.pdf]

S3 Table. Reaction list 2. It contains the list of reduced 28 reactions for mammalian CCM pathway under consideration. Here the reaction numbers correspond to the same serial numbers in S5 Table.

1. Glucose + ATP $\Rightarrow$ α-D glucose 6P + ADP
2. α-D glucose 6P $\Rightarrow$ β-D fructose 6P
3. β-D fructose 6P + ATP $\Rightarrow$ β-D fructose 1,6P_2 + ADP
4. β-D fructose 1,6P_2 $\Rightarrow$ D-Glyceraldehyde 3P + NADH
5. D-Glyceraldehyde 3P $\Rightarrow$ Glycerate 2P
6. Glycerate 2P $\Rightarrow$ Phosphoenolpyruvate (PEP)
7. Phosphoenolpyruvate (PEP) + ADP $\Rightarrow$ Pyruvate + ATP
8. Pyruvate $\Rightarrow$ Acetyl-CoA
9. Pyruvate $\Rightarrow$ Oxaloacetate
10. Oxaloacetate $\Rightarrow$ PEP
11. Oxaloacetate + Acetyl-CoA $\Rightarrow$ Citrate
12. Citrate $\Rightarrow$ Succinyl-CoA + NADH
13. Succinyl-CoA $\Rightarrow$ Oxaloacetate + NADH + GTP (ATP)
14. Oxaloacetate $\Rightarrow$ Succinyl-CoA
15. D-Glyceraldehyde 3P $\Rightarrow$ β-D fructose 1,6P_2
16. PEP $\Rightarrow$ Glycerate 2P
17. Glycerate 2P $\Rightarrow$ D-Glyceraldehyde 3P
18. β-D fructose 1,6P_2 $\Rightarrow$ β-D fructose 6P
19. β-D fructose 6P $\Rightarrow$ α-D glucose 6P
20. α-D glucose 6P $\Rightarrow$ Glucose
21. Pyruvate + NADH $\Rightarrow$ Lactate
22. Pyruvate $\Rightarrow$ Alanine
23. β-D fructose 6P $\Rightarrow$ β-D fructose 2,6P_2
24. β-D fructose 2,6P_2 $\Rightarrow$ β-D fructose 6P
25. α-D glucose 6P $\Rightarrow$ 6-phosphogluconate + NADPH
26. 6-phosphogluconate $\Rightarrow$ D-ribose-5P + NADPH
27. D-ribose-5P $\Rightarrow$ β-D fructose 6P
28. D-ribose-5P $\Rightarrow$ D-Glyceraldehyde 3P
