# Supplementary material for: Exploring the Altered Dynamics of Mammalian Central Carbon Metabolic Pathway in Cancer Cells: A Classical Control Theoretic Approach
Source: PLoS One. 2015 Sep 14;10(9):e0137728. doi: 10.1371/journal.pone.0137728 (PMC4569588; doi:10.1371/journal.pone.0137728)
Supplement: S4 Table — It contains the list of equations of reaction fluxes associated with 47 reactions from KEGG database for mammalian CCM pathway under consideration. Here the serial numbers correspond to the same reaction numbers in S2 Table. (PDF) [file pone.0137728.s007.pdf]

**S4 Table. Reaction equations 1.** It contains the list of equations of reaction fluxes associated with 47 reactions from KEGG database for mammalian CCM pathway under consideration. Here the serial numbers correspond to the same reaction numbers in S2 Table.

1. $v_1 = \frac{((K_{hexokinase} \cdot [glucose] \cdot [hexokinase]) \cdot (1+F_1 \cdot [Insulin]) \cdot (1+F_2 \cdot [ADP]))}{((K_{m_1}+[glucose]) \cdot (1+F_3 \cdot [\alpha Dglucose6P]))}$

2. $v_2 = \frac{(K_{glucose6phosphatase} \cdot [\alpha Dglucose6P] \cdot [glucose6phosphatase])}{(K_{m_2}+[\alpha Dglucose6P])}$

3. $v_3 = \frac{(K_{glucose6phosphateisomerase} \cdot [\beta Dglucose6P] \cdot [glucose6phosphateisomerase])}{(K_{m_3}+[\beta Dglucose6P])}$

4. $v_4 = \frac{(K_{glucose6phosphateisomerase} \cdot [\beta Dglucose6P] \cdot [glucose6phosphateisomerase])}{(K_{m_3}+[\beta Dglucose6P])}$

5. $v_5 = \frac{((K_{glucose6phosphateisomerase} \cdot [\alpha Dglucose6P] \cdot [glucose6phosphateisomerase]) \cdot (1+F_4 \cdot [NADPH]))}{(K_{m_5}+[\alpha Dglucose6P])}$

6. $v_6 = \frac{((K_{PFK1} \cdot [\beta Dfructose6P] \cdot [PFK1]) \cdot (1+F_5 \cdot [ADP]) \cdot (1+F_6 \cdot [\beta Dfructose2{,}6P_2]))}{((K_{m_6}+[\beta Dfructose6P]) \cdot (1+F_7 \cdot [ATP]) \cdot (1+F_8 \cdot [citrate]))}$

7. $v_7 = \frac{((K_{PFK2} \cdot [\beta Dfructose6P] \cdot [PFK2]) \cdot (1+F_9 \cdot [Insulin])}{(K_{m_7}+[\beta Dfructose6P])}$

8. $v_8 = \frac{((K_{FBPase2} \cdot [\beta Dfructose2{,}6P_2] \cdot [FBPase2]) \cdot (1+F_{10} \cdot [Glucagon]))}{(K_{m_8}+[\beta Dfructose2{,}6P_2])}$

9. $v_9 = \frac{(K_{aldolase} \cdot [\beta Dfructose1{,}6P_2] \cdot [aldolase])}{(K_{m_9}+[\beta Dfructose1{,}6P_2])}$

10. $v_{10} = \frac{(K_{aldolase} \cdot [\beta Dfructose1{,}6P_2] \cdot [aldolase])}{(K_{m_{10}}+[\beta Dfructose1{,}6P_2])}$

11. $v_{11} = \frac{(K_{triosephosphateisomerase} \cdot [GlyceroneP] \cdot [triosephosphateisomerase])}{(K_{m_{11}}+[GlyceroneP])}$

12. $v_{12} = \frac{(K_{glyceraldehyde3phosphatedehydrogenase} \cdot [DGlyceraldehyde3P] \cdot [glyceraldehyde3phosphatedehydrogenase])}{(K_{m_{12}}+[DGlyceraldehyde3P])}$

13. $v_{13} = \frac{(K_{phosphoglyceratekinase} \cdot [Glycerate1{,}3P_2] \cdot [phosphoglyceratekinase])}{(K_{m_{13}}+[Glycerate1{,}3P_2])}$

14. $v_{14} = \frac{(K_{phosphoglyceratemutase} \cdot [Glycerate3P] \cdot [phosphoglyceratemutase])}{(K_{m_{14}}+[Glycerate3P])}$

15. $v_{15} = \frac{(K_{enolase} \cdot [Glycerate2P] \cdot [enolase])}{(K_{m_{15}}+[Glycerate2P])}$

16. $v_{16} = \frac{((K_{pyruvatekinase} \cdot [PEP] \cdot [pyruvatekinase]) \cdot (1+F_{11} \cdot [ADP]) \cdot (1+F_{12} \cdot [\beta Dfructose6P]))}{((K_{m_{16}}+[PEP]) \cdot (1+F_{13} \cdot [ATP]) \cot(1+F_{14} \cdot [AcetylCoA]) \cdot (1+F_{15} \cdot [Alanine]) \cdot (1+F_{16} \cdot [longchainfattyacid]))}$

17. $v_{17} = \frac{(K_{phosphoenolpyruvatecarboxykinase} \cdot [oxaloacetate] \cdot [phosphoenolpyruvatecarboxykinase])}{(K_{m_{17}}+[oxaloacetate])}$

18. $v_{18} = \frac{((K_{pyruvatedehydrogenase} \cdot [pyruvatedehydrogenase] \cdot [pyruvate]) \cdot (1+F_{17} \cdot [Ca]))}{((1+F_{18} \cdot [AcetylCoA) \cdot (K_{m_{18}}+[pyruvate]) \cdot (1+F_{19} \cdot [NADH]) \cdot (1+F_{20} \cdot [ATP]) \cdot (1+F_{21} \cdot [longchainfattyacid]))}$

19. $v_{19} = \frac{((K_{pyruvatedehydrogenase} \cdot [pyruvatedehydrogenase] \cdot [2hydroxyethylThPP]) \cdot (1+F_{22} \cdot [Ca]))}{((1+F_{23} \cdot [AcetylCoA) \cdot (K_{m_{19}}+[2hydroxyethylThPP]) \cdot (1+F_{24} \cdot [NADH]) \cdot (1+F_{25} \cdot [ATP]) \cdot (1+F_{26} \cdot [longchainfattyacid]))}$

20. $v_{20} = \frac{((K_{pyruvatedehydrogenase} \cdot [pyruvatedehydrogenase] \cdot [SacetyldihydrolipoamideE]) \cdot (1+F_{27} \cdot [Ca]))}{((1+F_{28} \cdot [AcetylCoA) \cdot (K_{m_{20}}+[SacetyldihydrolipoamideE]) \cdot (1+F_{29} \cdot [NADH]) \cdot (1+F_{30} \cdot [ATP]) \cdot (1+F_{31} \cdot [longchainfattyacid]))}$

21. $v_{21} = \frac{((K_{pyruvatecarboxylase} \cdot [pyruvate] \cdot [pyruvatecarboxylase]) \cdot (1+F_{32} \cdot [AcetylCoA]))}{(K_{m_{21}}+[pyruvate])}$

22. $v_{22}^1 = \frac{((K_{citratesynthase} \cdot [citratesynthase] \cdot [AcetylCoA]) \cdot (1+F_{33} \cdot [ADP]))}{((1+F_{34} \cdot [citrate]) \cdot (K_{m_{22}}+[AcetylCoA]) \cdot (1+F_{35} \cdot [NADH]) \cdot (1+F_{36} \cdot [ATP]) \cdot (1+F_{37} \cdot [SuccinylCoA]))}$,
$v_{22}^2 = \frac{(K_{citratesynthase} \cdot [citratesynthase] \cdot [oxaloacetate])}{((K_{m_{22}}+[oxaloacetate]))}$

23. $v_{23} = \frac{(K_{aconitatehydratase} \cdot [citrate] \cdot [aconitatehydratase])}{(K_{m_{23}}+[citrate])}$

24. $v_{24} = \frac{(K_{aconitatehydratase} \cdot [CisAconitate] \cdot [aconitatehydratase])}{(K_{m_{24}}+[CisAconitate])}$

25. $v_{25} = \frac{((K_{isocitratedehydrogenase} \cdot [isocitratedehydrogenase] \cdot [Isocitrate]) \cdot (1+F_{38} \cdot [Ca]) \cdot (1+F_{39} \cdot [ADP]))}{((1+F_{40} \cdot [ATP]) \cdot (K_{m_{25}}+[Isocitrate]) \cdot (1+F_{41} \cdot [NADH]) \cdot (1+F_{42} \cdot [SuccinylCoA]))}$

26. $v_{26} = \frac{((K_{isocitratedehydrogenase} \cdot [isocitratedehydrogenase] \cdot [Oxalosuccinate]) \cdot (1+F_{43} \cdot [Ca]) \cdot (1+F_{44} \cdot [ADP]))}{((1+F_{45} \cdot [ATP]) \cdot (K_{m_{26}}+[Oxalosuccinate]) \cdot (1+F_{46} \cdot [NADH]) \cdot (1+F_{47} \cdot [SuccinylCoA]))}$

27. $v_{27} = \frac{((K_{2oxoglutaratedehydrogenase} \cdot [2oxoglutaratedehydrogenase] \cdot [2oxoglutarate]) \cdot (1+F_{48} \cdot [Ca]))}{((K_{m_{27}}+[2oxoglutarate]) \cdot (1+F_{49} \cdot [NADH]) \cdot (1+F_{50} \cdot [SuccinylCoA]))}$

28. $v_{28} = \frac{((K_{2oxoglutaratedehydrogenase} \cdot [2oxoglutaratedehydrogenase] \cdot [3carboxy1hydroxypropylThPP]) \cdot (1+F_{51} \cdot [Ca]))}{((K_{m_{28}}+[3carboxy1hydroxypropylThPP]) \cdot (1+F_{52} \cdot [NADH]) \cdot (1+F_{53} \cdot [SuccinylCoA]))}$

29. $v_{29} = \frac{((K_{2oxoglutaratedehydrogenase} \cdot [2oxoglutaratedehydrogenase] \cdot [SsuccinyldihydrolipoamideE]) \cdot (1+F_{54} \cdot [Ca]))}{((K_{m_{29}}+[SsuccinyldihydrolipoamideE]) \cdot (1+F_{55} \cdot [NADH]) \cdot (1+F_{56} \cdot [SuccinylCoA]))}$

30. $v_{30} = \frac{(K_{succinylCoAsynthetase} \cdot [SuccinylCoA] \cdot [succinylCoAsynthetase])}{(K_{m_{30}}+[SuccinylCoA])}$

31. $v_{31} = \frac{(K_{succinatedehydrogenase} \cdot [Succinate] \cdot [succinatedehydrogenase])}{(K_{m_{31}}+[Succinate])}$

32. $v_{32} = \frac{(K_{fumaratehydratase} \cdot [Fumarate] \cdot [fumaratehydratase])}{(K_{m_{32}}+[Fumarate])}$

33. $v_{33} = \frac{(K_{malatedehydrogenase} \cdot [(S)malate] \cdot [malatedehydrogenase])}{(K_{m_{33}}+[(S)malate])}$

34. $v_{34} = \frac{(K_{glucose6phosphateisomerase} \cdot [\alpha Dglucose6P] \cdot [glucose6phosphateisomerase])}{(K_{m_{34}}+[\alpha Dglucose6P])}$

35. $v_{35} = \frac{((K_{glucose6phosphatedehydrogenase} \cdot [\beta Dglucose6P] \cdot [glucose6phosphatedehydrogenase]))}{((K_{m_{35}}+[\beta Dglucose6P]) \cdot (1+F_{57} \cdot [NADPH]))}$

36. $v_{36} = \frac{(K_{6phosphogluconolactonase} \cdot [DGlucono1{,}5lactone6P] \cdot [6phosphogluconolactonase])}{(K_{m_{36}}+[DGlucono1{,}5lactone6P])}$

37. $v_{37} = \frac{(K_{6phosphogluconatedehydrogenase} \cdot [6phosphogluconate] \cdot [6phosphogluconatedehydrogenase])}{(K_{m_{37}}+[6phosphogluconate])}$

38. $v_{38} = \frac{(K_{ribose5phosphateisomerase} \cdot [Ribulose5phosphate] \cdot [ribose5phosphateisomerase])}{(K_{m_{38}}+[Ribulose5phosphate])}$

39. $v_{39} = \frac{(K_{ribulosephosphate3epimerase} \cdot [Ribulose5phosphate] \cdot [ribulosephosphate3epimerase])}{(K_{m_{39}}+[Ribulose5phosphate])}$

40. $v_{40}^1 = \frac{(K_{transketolase1} \cdot [Dribose5P] \cdot [transketolase1])}{(K_{m_{40}}+[Dribose5P])}$,
$v_{40}^2 = \frac{(K_{transketolase1} \cdot [transketolase1] \cdot [Xylulose5phosphate])}{((K_{m_{40}}+[Xylulose5phosphate]))}$

41. $v_{41} = \frac{(K_{aldolase'} \cdot [DGlyceraldehyde3P] \cdot [aldolase'])}{(K_{m_{41}}+[DGlyceraldehyde3P])}$

42. $v_{42} = \frac{(K_{fructose1,6bisphosphatase1} \cdot [\beta Dfructose1{,}6P_2] \cdot [fructose1{,}6bisphosphatase1])}{(K_{m_{42}}+[\beta Dfructose1{,}6P_2])}$

43. $v_{43} = \frac{(K_{glucose6phosphateisomerase'} \cdot [\beta Dfructose6P] \cdot [glucose6phosphateisomerase'])}{(K_{m_{43}}+[\beta Dfructose6P])}$

44. $v_{44}^{1} = \frac{(K_{transaldolase} \cdot [Sedoheptulose7phosphate] \cdot [transaldolase])}{(K_{m_{44}} + [Sedoheptulose7phosphate])}$,
$v_{44}^{2} = \frac{(K_{transaldolase} \cdot [transaldolase] \cdot [DGlyceraldehyde3P])}{((K_{m_{44}} + [DGlyceraldehyde3P]))}$

45. $v_{45}^{1} = \frac{(K_{transketolase1} \cdot [Xylulose5phosphate] \cdot [transketolase1])}{(K_{m_{45}} + [Xylulose5phosphate])}$,
$v_{45}^{2} = \frac{(K_{transketolase1} \cdot [transketolase1] \cdot [Erythrose4phosphate])}{((K_{m_{45}} + [Erythrose4phosphate]))}$

46. $v_{46} = \frac{(K_{lactatedehydrogenase} \cdot [pyruvate] \cdot [lactatedehydrogenase])}{(K_{m_{46}} + [pyruvate])}$

47. $v_{47} = \frac{(K_{alaninetransaminase} \cdot [pyruvate] \cdot [alaninetransaminase])}{(K_{m_{47}} + [pyruvate])}$

---

**Note 1**. We have considered reaction number 22 (S1 Table) as Acetyl-CoA $\Rightarrow$ Citrate with flux $v_{22}^{1}$. Oxaloacetate consumption in this particular reaction has been taken as a separate reaction with flux $v_{22}^{2}$. In this scenario, the reaction rate constant $K_{citratesynthase}$ and Michaelis constant $K_{m_{11}}$ are same for both the equations corresponding to the reaction fluxes $v_{22}^{1}$ and $v_{22}^{2}$. Reaction numbers 40,44 and 45 (S1 Table) have been treated similarly.
**Note 2**. $Net\,ATP$ = initial concentration of $ATP$ − ($ATP$ consumption in reaction number 1 (S1 Table) *i.e.* proportionate to $\alpha$-D glucose 6P production) − ($ATP$ consumption in reaction number 6 (S1 Table) *i.e.* proportionate to $\beta$-D fructose 1,6$P_2$ production) + ($ATP$ production from reaction number 13 (S1 Table) *i.e.* proportionate to Glycerate 3P production) + ($ATP$ production from reaction number 16 (S1 Table) *i.e.* proportionate to pyruvate production) + ($ATP$ production from reaction number 30 (S1 Table) *i.e.* proportionate to Succinate production). The same technique has been applied to calculate $Net\,ADP$, $Net\,NADH$ and $Net\,NADPH$.
