# Supplementary material for: Exploring the Altered Dynamics of Mammalian Central Carbon Metabolic Pathway in Cancer Cells: A Classical Control Theoretic Approach
Source: PLoS One. 2015 Sep 14;10(9):e0137728. doi: 10.1371/journal.pone.0137728 (PMC4569588; doi:10.1371/journal.pone.0137728)
Supplement: S5 Table — It contains the list of equations of reaction fluxes associated with reduced 28 reactions from KEGG database for mammalian CCM pathway under consideration. Here the serial numbers correspond to the same reaction numbers in S3 Table. (PDF) [file pone.0137728.s008.pdf]

S5 Table. Reaction equations 2. It contains the list of equations of reaction fluxes associated with reduced 28 reactions from KEGG database for mammalian CCM pathway under consideration. Here the serial numbers correspond to the same reaction numbers in S3 Table.

1. $v_1 = \frac{((K_{hexokinase}\cdot[glucose]\cdot[hexokinase])\cdot(1+F_1\cdot[Insulin])\cdot(1+F_2\cdot[ADP]))}{((K_{m_1}+[glucose])\cdot(1+F_3\cdot[\alpha Dglucose6P]))}$

2. $v_2 = \frac{((K_{glucose6phosphateisomerase}\cdot[\alpha Dglucose6P]\cdot[glucose6phosphateisomerase])\cdot(1+F_4\cdot[NADPH]))}{(K_{m_2}+[\alpha Dglucose6P])}$

3. $v_3 = \frac{((K_{PFK1}\cdot[\beta Dfructose6P]\cdot[PFK1])\cdot(1+F_5\cdot[ADP])\cdot(1+F_6\cdot[\beta Dfructose2{,}6P_2]))}{((K_{m_3}+[\beta Dfructose6P])\cdot(1+F_7\cdot[ATP])\cdot(1+F_8\cdot[citrate]))}$

4. $v_4 = \frac{(K_{aldolase}\cdot[\beta Dfructose1{,}6P_2]\cdot[aldolase])}{(K_{m_4}+[\beta Dfructose1{,}6P_2])}$

5. $v_5 = \frac{(K_{phosphoglyceratemutase}\cdot[DGlyceraldehyde3P]\cdot[phosphoglyceratemutase])}{(K_{m_5}+[DGlyceraldehyde3P])}$

6. $v_6 = \frac{(K_{enolase}\cdot[Glycerate2P]\cdot[enolase])}{(K_{m_6}+[Glycerate2P])}$

7. $v_7 = \frac{((K_{pyruvatekinase}\cdot[PEP]\cdot[pyruvatekinase])\cdot(1+F_9\cdot[ADP])\cdot(1+F_{10}\cdot[\beta Dfructose6P]))}{((K_{m_7}+[PEP])\cdot(1+F_{11}\cdot[ATP])\cot(1+F_{12}\cdot[AcetylCoA])\cdot(1+F_{13}\cdot[Alanine])\cdot(1+F_{14}\cdot[longchainfattyacid]))}$

8. $v_8 = \frac{((K_{pyruvatedehydrogenase}\cdot[pyruvatedehydrogenase]\cdot[pyruvate])\cdot(1+F_{15}\cdot[Ca]))}{((1+F_{16}\cdot[AcetylCoA)\cdot(K_{m_8}+[pyruvate])\cdot(1+F_{17}\cdot[NADH])\cdot(1+F_{18}\cdot[ATP])\cdot(1+F_{19}\cdot[longchainfattyacid]))}$

9. $v_9 = \frac{((K_{pyruvatecarboxylase}\cdot[pyruvate]\cdot[pyruvatecarboxylase])\cdot(1+F_{20}\cdot[AcetylCoA]))}{(K_{m_9}+[pyruvate])}$

10. $v_{10} = \frac{(K_{phosphoenolpyruvatecarboxykinase}\cdot[oxaloacetate]\cdot[phosphoenolpyruvatecarboxykinase])}{(K_{m_{10}}+[oxaloacetate])}$

11. $v_{11}^1 = \frac{((K_{citratesynthase}\cdot[citratesynthase]\cdot[AcetylCoA])\cdot(1+F_{21}\cdot[ADP]))}{((1+F_{22}\cdot[citrate])\cdot(K_{m_{11}}+[AcetylCoA])\cdot(1+F_{23}\cdot[NADH])\cdot(1+F_{24}\cdot[ATP])\cdot(1+F_{25}\cdot[SuccinylCoA]))}$,
$v_{11}^2 = \frac{(K_{citratesynthase}\cdot[citratesynthase]\cdot[oxaloacetate])}{((K_{m_{11}}+[oxaloacetate]))}$

12. $v_{12} = \frac{((K_{2oxoglutaratedehydrogenase}\cdot[2oxoglutaratedehydrogenase]\cdot[citrate])\cdot(1+F_{26}\cdot[Ca])\cdot(1+F_{27}\cdot[ADP]))}{((1+F_{28}\cdot[ATP])\cdot(K_{m_{12}}+[citrate])\cdot(1+F_{29}\cdot[NADH])\cdot(1+F_{30}\cdot[SuccinylCoA]))}$

13. $v_{13} = \frac{(K_{malatedehydrogenase}\cdot[SuccinylCoA]\cdot[malatedehydrogenase])}{(K_{m_{13}}+[SuccinylCoA])}$

14. $v_{14} = \frac{(K_{succinateCoAligase}\cdot[oxaloacetate]\cdot[succinateCoAligase])}{(K_{m_{14}}+[oxaloacetate])}$

15. $v_{15} = \frac{(K_{aldolase'}\cdot[DGlyceraldehyde3P]\cdot[aldolase'])}{(K_{m_{15}}+[DGlyceraldehyde3P])}$

16. $v_{16} = \frac{(K_{enolase'}\cdot[PEP]\cdot[enolase'])}{(K_{m_{16}}+[PEP])}$

17. $v_{17} = \frac{(K_{glyceraldehyde3phosphatedehydrogenase}\cdot[Glycerate2P]\cdot[glyceraldehyde3phosphatedehydrogenase])}{(K_{m_{17}}+[Glycerate2P])}$

18. $v_{18} = \frac{(K_{fructose1,6bisphosphatase1}\cdot[\beta Dfructose1{,}6P_2]\cdot[fructose1{,}6bisphosphatase1])}{(K_{m_{18}}+[\beta Dfructose1{,}6P_2])}$

19. $v_{19} = \frac{(K_{glucose6phosphateisomerase'}\cdot[\beta Dfructose6P]\cdot[glucose6phosphateisomerase'])}{(K_{m_{19}}+[\beta Dfructose6P])}$

20. $v_{20} = \frac{(K_{glucose6phosphatase}\cdot[\alpha Dglucose6P]\cdot[glucose6phosphatase])}{(K_{m_{20}}+[\alpha Dglucose6P])}$

21. $v_{21} = \frac{(K_{lactatedehydrogenase} \cdot [pyruvate] \cdot [lactatedehydrogenase])}{(K_{m_{21}} + [pyruvate])}$

22. $v_{22} = \frac{(K_{alaninetransaminase} \cdot [pyruvate] \cdot [alaninetransaminase])}{(K_{m_{22}} + [pyruvate])}$

23. $v_{23} = \frac{((K_{PFK2} \cdot [\beta Dfructose6P] \cdot [PFK2]) \cdot (1 + F_{31} \cdot [Insulin])}{(K_{m_{23}} + [\beta Dfructose6P])}$

24. $v_{24} = \frac{((K_{FBPase2} \cdot [\beta Dfructose2{,}6P_2] \cdot [FBPase2]) \cdot (1 + F_{32} \cdot [Glucagon]))}{(K_{m_{24}} + [\beta Dfructose2{,}6P_2])}$

25. $v_{25} = \frac{((K_{glucose6phosphatedehydrogenase} \cdot [\alpha Dglucose6P] \cdot [glucose6phosphatedehydrogenase]))}{((K_{m_{25}} + [\alpha Dglucose6P]) \cdot (1 + F_{33} \cdot [NADPH]))}$

26. $v_{26} = \frac{(K_{ribose5phosphateisomerase} \cdot [6phosphogluconate] \cdot [ribose5phosphateisomerase])}{(K_{m_{26}} + [6phosphogluconate])}$

27. $v_{27} = \frac{(K_{transaldolase} \cdot [Dribose5P] \cdot [transaldolase])}{(K_{m_{27}} + [Dribose5P])}$

28. $v_{28} = \frac{(K_{transketolase1} \cdot [Dribose5P] \cdot [transketolase1])}{(K_{m_{28}} + [Dribose5P])}$

Note 1. We have considered reaction number 11 (S2 Table) as Acetyl-CoA $\Rightarrow$ Citrate with flux v_{11}^1. Oxaloacetate consumption in this particular reaction has been taken as a separate reaction with flux v_{11}^2. In this scenario, the reaction rate constant $K_{citratesynthase}$ and Michaelis constant $K_{m_{11}}$ are same for both the equations corresponding to the reaction fluxes v_{11}^1 and v_{11}^2.
Note 2. $Net\,ATP$ = initial concentration of ATP − (ATP consumption in reaction number 1 (S2 Table) *i.e.* proportionate to α-D glucose 6P production) − (ATP consumption in reaction number 3 (S2 Table) *i.e.* proportionate to β-D fructose 1,6P_2 production) + (ATP production from reaction number 7 (S2 Table) *i.e.* proportionate to pyruvate production) + (ATP production from reaction number 13 (S2 Table) *i.e.* proportionate to oxaloacetate production). The same technique has been applied to calculate $Net\,ADP$, $Net\,NADH$ and $Net\,NADPH$.
