# Supplementary material for: Exploring the Altered Dynamics of Mammalian Central Carbon Metabolic Pathway in Cancer Cells: A Classical Control Theoretic Approach
Source: PLoS One. 2015 Sep 14;10(9):e0137728. doi: 10.1371/journal.pone.0137728 (PMC4569588; doi:10.1371/journal.pone.0137728)
Supplement: S6 Table — Here, the simulation parameters for the proposed CCM pathway model are given. (PDF) [file pone.0137728.s009.pdf]

S6 Table. Simulation parameters. Here, the simulation parameters for the proposed CCM pathway model are given.

Simulation Time		
	Start Time	10 s
	Stop Time	30000 s
Solver option	Type	Variable-step
	Solver	Ode45(Dormand-Prince)
	Max step size	Auto
	Min step size	Auto
	Relative tolerance	1×10^{-3}
	Initial step size	Auto
	Absolute tolerance	Auto
	Shape preservation	Disable all
	Number of consecutive min steps	1
Zero-crossing options	Zero-crossing control	Use local settings
	Algorithm	Nonadaptive
	Time tolerance	$10\times128\times$eps
	Signal thresold	Auto
	Number of consecutive zero crossings	1000
